# Miniaturized high-throughput conversion of fungal strain collections into chemically characterized extract libraries for antimicrobial discovery

**DOI:** 10.3389/fchem.2025.1630332

**Published:** 2025-07-02

**Authors:** Alexandre Bory, Alexandre Luscher, Nicole Lecoultre, Thilo Köhler, Sylvain Schnee, Katia Gindro, Jean-Luc Wolfender

**Affiliations:** ^1^ School of Pharmaceutical Sciences, University of Geneva, Centre Medical Universitaire, Geneva, Switzerland; ^2^ Institute of Pharmaceutical Sciences of Western Switzerland, University of Geneva, Centre Medical Universitaire, Geneva, Switzerland; ^3^ Department of Microbiology and Molecular Medicine, University of Geneva, Geneva, Switzerland; ^4^ Mycology Group, Research Department Plant Protection, Agroscope, Nyon, Switzerland

**Keywords:** drug discovery, fungal strains, metabolomics, antimicrobial, ESKAPE, AMR, FLECS-96

## Abstract

Natural products remain vital to drug discovery, with fungi representing an underexplored source of bioactive compounds. Despite advances in LC-MS-based metabolomics that facilitate dereplication and chemical profiling of natural extracts, the rate of new discoveries has not significantly increased. This stagnation may be attributed to the laborious process of culturing and extracting large microbial collections. At the same time, rising antimicrobial resistance, particularly among ESKAPE pathogens, highlights the urgent need for new scaffolds. To address these challenges, we developed FLECS-96 (Fungal Library Extract Conversion and Screening in 96-well plate format), a high-throughput platform that efficiently transforms fungal strains into chemically characterized extract libraries. FLECS-96 combines miniaturized fungal liquid culture with streamlined sample preparation, systematic metabolomic profiling, and biological evaluation of the extracts. Here, we describe the development and validation of this workflow, and demonstrate its utility through the rapid identification of compounds active against *S. aureus*. FLECS-96 provides a scalable solution to accelerate antimicrobial lead discovery from fungal sources.

## 1 Introduction

Antimicrobial resistance (AMR) is a growing global health crisis, threatening the effectiveness of antibiotics and threaten decades of medical progress (Shallcross and Davies, 2014; Ventola, 2015). The ESKAPE pathogens, in particular, have acquired resistance to most first-line treatments, and projections estimate AMR could cause 10 million deaths annually by 2050 (O’Neill, 2014; 2016). The COVID-19 pandemic has further exacerbated this issue by increasing antibiotic use and disrupting drug development pipelines (Saleem et al., 2019).

Pharmaceutical investment in antibiotic discovery has declined due to weak economic incentives, leaving academic and small enterprises to lead innovation (Böttcher et al., 2022). In this context, natural products (NPs), especially those derived from fungi, remain essential for drug discovery due to their structural diversity and potent bioactivities (Borel, 2002; Berdy, 2012; Lewis, 2013; Newman and Cragg 2020). Fungal metabolites have yielded immunosuppressants, antifungals, anticancer agents, and antibiotics; often with superior bacterial penetration compared to synthetic compounds (Rupshikha et al., 2024).

Despite the estimated millions of fungal species, only a fraction has been taxonomically described and chemically investigated (Blackwell, 2011; Wu et al., 2019; Phukhamsakda et al., 2022), highlighting a vast unexplored reservoir of potential drug leads (Boruta, 2018).

Recent advances in mass spectrometry based metabolomics have accelerated the dereplication and annotation of microbial metabolites (Alarcon-Barrera et al., 2022; Bauermeister et al., 2022). Tools like GNPS (Wang et al., 2016), SIRIUS (Dührkop et al., 2019), and FBMN (Nothias et al., 2020) have enhanced data interpretation, enabling researchers to prioritize novel compounds more efficiently.

Nevertheless, extract production remains a major bottleneck. Traditional culture and extraction methods are slow and resource-intensive. To address this, we developed FLECS-96 (**F**ungal **L**ibrary **E**xtract **C**onversion and **S**creening in **96**-well format), a high-throughput platform for generating assay-ready fungal extracts. It integrates miniaturized cultivation, extraction, and sample preparation with metabolomic profiling, reducing manual handling and preserving extract integrity.

Our system supports downstream antimicrobial screening via phenotypic assays (Swenson et al., 2011), enabling rapid identification of active compounds against clinically relevant pathogens.

To our knowledge, this is the first integrated workflow tailored for fungal NP-based antibiotic discovery. By streamlining extract preparation and bioactivity screening, FLECS-96 offers a scalable solution to accelerate the identification of novel antimicrobial agents.

## 2 Results

FLECS-96, a high-throughput screening workflow, was developed to rapidly convert a fungal strain collection into a chemically characterized extract library suitable for antimicrobial screening. This approach utilizes a miniaturized 96-well plate format throughout the entire process, enabling efficient screening of numerous fungal strains, defined thereafter as “small-scale”.

First, the feasibility of conducting liquid fungal cultures directly in deep-well plates was evaluated (2.1.1). Several extraction protocols were then assessed and validated using known representative antibiotics, with monitoring by UHPLC-HRMS/MS (2.1.2). A plate of fungal extracts from selected strains was subsequently generated (2.1.3) to confirm metabolite production and evaluate potential cross-contamination (2.1.4). Finally, to assess the sensitivity of the workflow for antimicrobial detection, a known antibiotic was spiked into a complex extract, and both the bioactivity and MS detection thresholds were determined (2.1.5).

As a proof of concept, the developed methodology was applied to two distinct sets of fungal strains for bioactivity evaluation, metabolomic profiling, and comprehensive data analysis aimed at revealing potential antimicrobial compounds (1.2).

### 2.1 Development and validation of an effective workflow: culture–extraction and analysis

#### 2.1.1 Evaluation of small-scale fungal liquid culture

The feasibility of small-scale fungal liquid culture was evaluated using 96-well plates containing Potato Dextrose Broth (PDB), a generic medium selected for broad compatibility with diverse fungal strains. Stored strains from the Agroscope Mycoscope collection were revived on agar plates for 7 days, followed by inoculation in liquid media using small agar plugs. To maintain the 96-well plate format while maximizing broth volume, cultures were incubated using deep-well plates and the Duetz system (Duetz, 2007). Growth was monitored through regular visual inspection, which confirmed sufficient mycelial development after 2 weeks of cultivation, enabling subsequent extraction steps.

#### 2.1.2 Selection and assessment of extraction protocols using a representative set of antibiotics

##### 2.1.2.1 Selection of small-scale extraction protocols for fungal liquid cultures

Three extraction methods compatible with 96-well plates were selected for *in situ* extraction of fungal metabolites: Solid Phase Extraction (SPE), Supported Liquid Extraction (SLE), and QuEChERS (Quick, Easy, Cheap, Effective, Rugged, and Safe). The QuEChERS methodology, originally developed for pesticide analysis (Anastassiades et al., 2003), was adapted for its versatility in extracting diverse compounds, including antibiotics, from complex matrices. QuEChERS protocols typically involve an acetonitrile-water extraction followed by a clean-up using materials such as primary secondary amine (PSA) and/or C18 silica. Four QuEChERS variations were selected based on matrix type and targeted metabolites (Desmarchelier et al., 2018; He et al., 2018; Ajibola et al., 2020; Zhu et al., 2021). Each protocol was adapted to fit the well volume (2 mL) with approx. 1.5 mL of fungal broth supernatant available after culture. Detailed protocols and mixture compositions are provided in Supplementary Material.

SPE with C18 bonded silica was chosen for its commercial availability in 96-well format and ability to retain apolar compounds. The protocol involves classical cartridge conditioning, water washing, and methanol elution. SLE, also commercially available in 96-well format, was included for its simplicity, requiring only loading and EtOAc elution steps; thus, making it an attractive option for high-throughput screening.

##### 2.1.2.2 Experimental design for extraction protocols assessment

A mixture of antibiotics, with logP values ranging from −1 to +6 (Table 1), was used to simultaneously assess extraction efficiency and reproducibility across different conditions. This polarity range was consistent with that expected for targeted fungal secondary metabolites. These compounds, selected according to Lipinski’s Rule of Five (Lipinski et al., 1997), exhibited retention times between 0.9 and 4.5 min under a generic UHPLC-HRMS/MS profiling method with a 7-min linear gradient (5%–100% B) (see Section 4.9.3).

**TABLE 1:** Antibiotics used for extraction protocol assessment with their LogP Values and retention time (RT) in the generic reverse phase UHPLC-HRMS/MS chromatographic method described in chapter 5.9.3.

N°	Name	LogP	RT (min)
1	Lincomycin	−0.9	0.95
2	Ampicillin	0.3	1.09
3	Tetracycline	−1.3	1.28
4	Azithromycin monohydrate	1.9	1.77
5	Oxolinic acid	1.4	1.87
6	Oxacillin	1.9	2.76
7	Clarithromycin	2.4	2.83
8	Josamycin	3.0	3.01
9	Rifampicin	4.3	3.56
10	Novobiocin	4.1	4.19
11	Fusidic acid	5.7	4.52

A 96-well plate was designed to orthogonally test these six extraction protocols across three representative liquid matrices: water, culture medium (PDB), and a fungal broth filtrate. The latter was included to closely mimic conditions encountered during the extraction of fungal supernatants. This design served two main purposes: 1) to evaluate the efficacy of each extraction protocol across different matrices, and 2) to assess the feasibility of small-scale extractions directly in a high-throughput 96-well format (Supplementary Figure S1).

Each matrix was spiked with the mixture of antibiotics (5 ppm each) prior to extraction. This strategy ensured that all extraction protocols were challenged with analytes covering a broad polarity range, reflecting the chemical diversity expected in fungal metabolomic studies.

Antibiotic stock solutions were prepared in ${\text{H}}_{2}\text{O}$ /ACN (90:10, v/v) and serially diluted from 0 to 100 ppm. Calibration curves were constructed for each antibiotic using the same UHPLC-HRMS method applied throughout the study (see Supplementary Material), ensuring consistency in detection and quantification. These calibration curves were then used to calculate recovery rates from the spiked matrices after extraction and analysis.

##### 2.1.2.3 Assessment of small-scale extraction protocols

**FIGURE 1:**
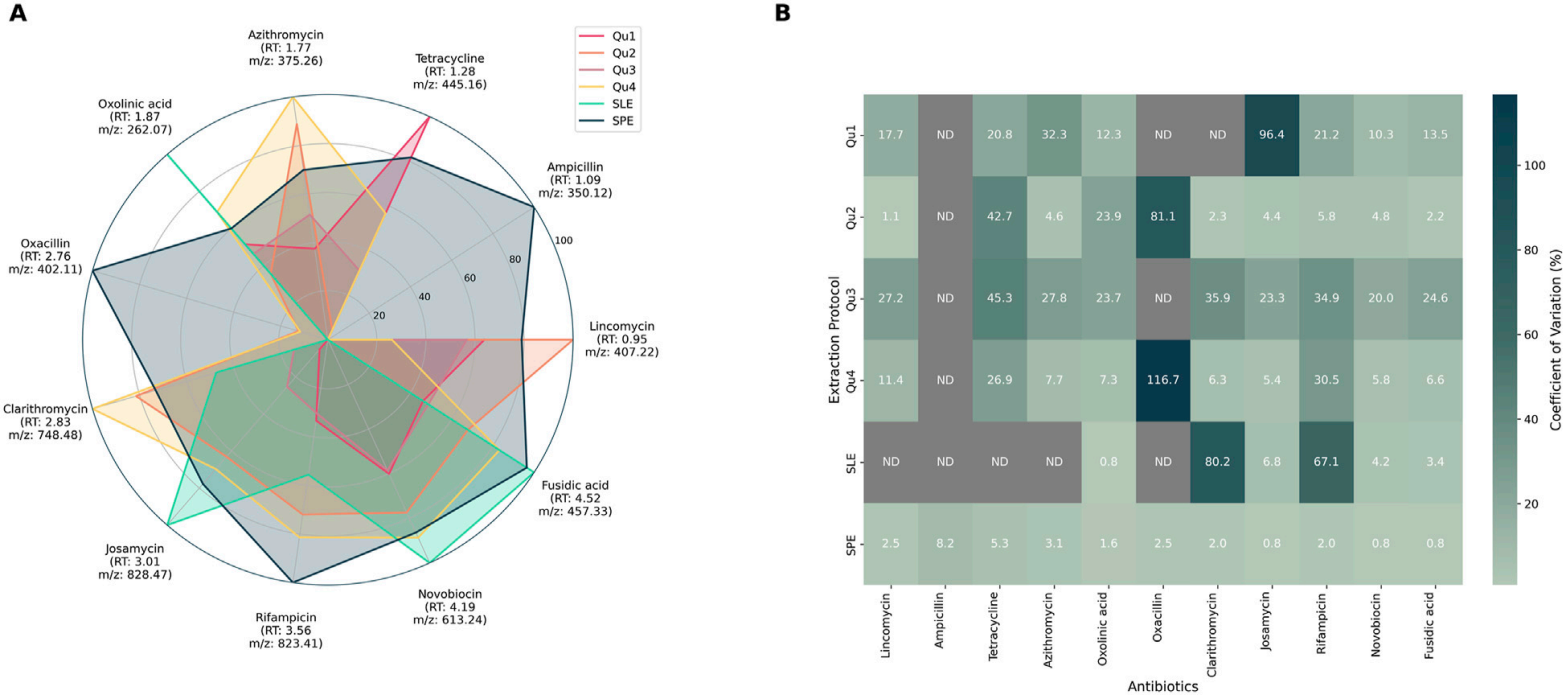
Recovery rates **(A)** and coefficient of variation **(B)** obtained for each antibiotic extraction using specific protocols in the representative fungal broth. **(A)**. Antibiotic recovery rates obtained for each extraction method: Data were normalized per antibiotic. Each antibiotic is displayed around the spider plot with its corresponding retention time (RT) and the *m/z* value used for quantification. For each combination (antibiotic:extraction protocol), the mean value of replicates was used. Each color corresponds to an extraction method: QuEChERS (Qu1-4), Supported Liquid Extraction (SLE), and Solid Phase Extraction (SPE). **(B)**. Heat map of the variability results expressed as the coefficient of variation in % for each antibiotic and extraction protocol. ND: Not detected.

Extraction performance was evaluated by calculating both recovery rates and variability across replicates. Recovery was determined by integrating single-ion traces and applying protocol-specific concentration factors. Variability was quantified using the coefficient of variation (CV) across replicates for each antibiotic-extraction protocol combination. Results obtained from the fungal broth filtrate—the most representative matrix—are shown in Figure 1, where normalized recovery rates (per antibiotics) are visualized as a spider plot (Figure 1A) and replicate variability is illustrated as a heatmap (Figure 1B). Results for the two other matrices are available in Supplementary data (Supplementary Figures S3, S4).

Among tested protocols, SPE demonstrated the best overall performance, with high recovery rates, excellent reproducibility (CV ≤ 10%), and rapid processing. In contrast, QuEChERS was less scalable due to labor-intensive and error-prone manual salt additions, which introduced variability across wells. Although supported liquid extraction (SLE) was scalable, its high variability made it unsuitable for further use (see Figure 1B).

#### 2.1.3 Development of SPE fungal metabolites enrichment protocol

With SPE established as a robust method for extracting a wide variety of spiked antibiotics, we focused on developing a protocol for extracting metabolites from small-scale fungal culture.

The first step was to effectively disrupt the fungal mycelium directly in the deep-well plate after culture in liquid medium. Tissue Lyser combined with beads are commonly used for mechanical tissue disruption (Goldberg, 2021). Tungsten beads (one per well) successfully disrupted the mycelium, while glass beads proved ineffective. To prevent cross-contamination, a silicone mat was used to seal the plate. A test using a dye in alternating wells confirmed the setup’s effectiveness, as no detectable contamination was observed.

The protocol was applied to a representative set of fourteen fungal strains, including eight *Fusarium* sp., two *Botrytis cinerea*, two *Chaetomium globosum*, one *Coprinellus* sp., and one *Trichoderma* sp. This taxonomically diverse selection provided a broad context for metabolomic analysis. Multiple species from the same genus were intentionally included to assess the method’s ability to distinguish closely related strains based on their metabolomic profiles, allowing evaluation of both inter-genus and intra-genus diversity.

To evaluate the consistency of metabolites production in independent biological replicates, each strain was inoculated in randomized replicates (n = 6) into deep wells containing culture media (ABO-P26). After 14 days of growth, extracts were generated using the SPE-based enrichment protocol.

In brief, a tungsten bead was added to each well and the plate was homogenized with a TissueLyser. After an hour of centrifugation ensuring complete sedimentation, the clear supernatant was loaded onto the SPE cartridges. The protocol included a systematic water wash to eliminate highly polar compounds and elution with methanol allowed medium-polarity metabolites to flow out of the SPE cartridge while retaining lipophilic cell-wall constituents like ergosterol (Pasanen et al., 1999).

Methanol eluates were collected and dried, typically yielding around 0.5 mg of extract per well. The use of individually pre-weighed wells enabled an estimation of extract mass after drying, which in turn allowed standardization at 5 mg/mL in DMSO. This approach ensured consistent extract concentrations across samples, thereby facilitating reliable comparison in subsequent metabolomics and bioassay steps. DMSO was chosen for its ability to solubilize a broad range of analytes and its compatibility with both metabolomics and bioassays.

In summary, our developed 96-well plate-based protocol streamlines the process from fungal strains to extracts. This approach yielded a mother plate of extracts at 5 mg/mL in DMSO, compatible with metabolomic analyses and biological screening.

#### 2.1.4 Evaluation of metabolites production and strain-specificity by metabolomics

Metabolite profiling of each individual well was performed using data-dependent UHPLC-CAD-HRMS/MS (Orbitrap platform) to evaluate the metabolites produced by the fungal strains at small scale. HRMS/MS data obtained for the entire plate were processed using MzMine (Schmid et al., 2023; Heuckeroth et al., 2024), generating an aligned ${\text{MS}}^{1}$ feature table for all extracts and a corresponding ${\text{MS}}^{2}$ spectral file (see Materials and Methods for details).

**FIGURE 2:**
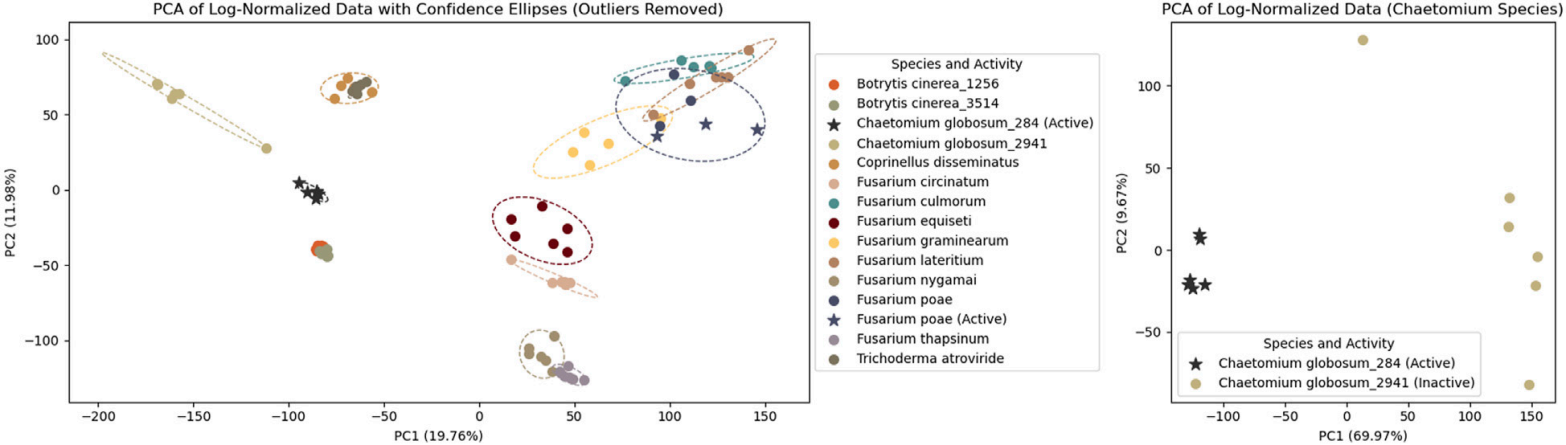
PCA of log-normalized HRMS analysis of 14 fungal strains cultured in microplates. Each dot represents an extract, with colors corresponding to different fungal strains. Outliers (n = 3) and blanks (n = 12) were removed. Stars indicate extracts active against *Staphylococcus aureus*. The left panel shows all extracts, while the right panel focuses on two *Chaetomium globosum* strains: one active (stars) and one non-active (dots). The middle section lists the species, their strain IDs (for multiple strains of the same species), and the results of antimicrobial activity tests against *S. aureus*.

To assess replicate reproducibility and strain specificity, a multivariate data analysis (PCA) was performed on the log-normalized ${\text{MS}}^{1}$ feature table which shows consistent clustering based on the sample types. Three out of 84 extracts were identified as outliers and excluded from further analysis based on individual chromatographic profile inspection. The analysis revealed clustering by fungal strain, with PC1 and PC2 explaining approximately 30% of the dataset variance (see left panel of Figure 2). This clustering indicated distinct chemical compositions for each strain and high reproducibility among replicates from the culture to the extraction. Additionally, strains of the same species, such as the two *B. cinerea*, clustered together, demonstrating the robustness of the protocol and the suitability of MS-based metabolomics in this context. In some cases, strain specificity within the same species was observed, as detailed in the example of *C. globosum* below.

For an in depth comparison of strain metabolites, ${\text{MS}}^{2}$ spectra associated with ${\text{MS}}^{1}$ feature used in the PCA were further organized and annotated using the GNPS molecular networking workflow (Aron et al., 2020). The resulting FBMN, which allows for the visualization and grouping of structurally similar features, contained 3507 nodes, 5097 edges and 2202 connected components. Of these, 91 nodes were successfully annotated through experimental spectral matching in the GNPS workflow. Some clusters were non-specific, related to culture media or ubiquitous fungal molecules like 2,5-diketopiperazines (2,5-DKPs), which are widespread in fungi (Wang et al., 2017). Other clusters exhibited species specificity, such as a chaetoglobosins cluster for *C. globosum* and a fumonisins cluster for *Fusarium poae*, consistent with previous studies (Sekita et al., 1981; Jiao et al., 2004; Thrane et al., 2004). In particular, chaetoglobosins, including Chaetoglobosin A, are cytochalasans metabolites from *C. globosum* (Scherlach et al., 2010; Jiao et al., 2004; Xue et al., 2012), confirming the specificity of the observed metabolomic profiles.

#### 2.1.5 Assessment of active compounds detection’s thresholds in complex extracts

The bioassay’s threshold and its compatibility with both fungal extracts and MS-based detection was evaluated. The goal was to determine whether antimicrobial compounds responsible for an extract’s activity could be detected by mass spectrometry. *Staphylococcus aureus*, a clinically relevant pathogen and member of the ESKAPE group of pathogens, was used as the test organism to validate the method’s applicability for antimicrobial discovery.

**FIGURE 3:**
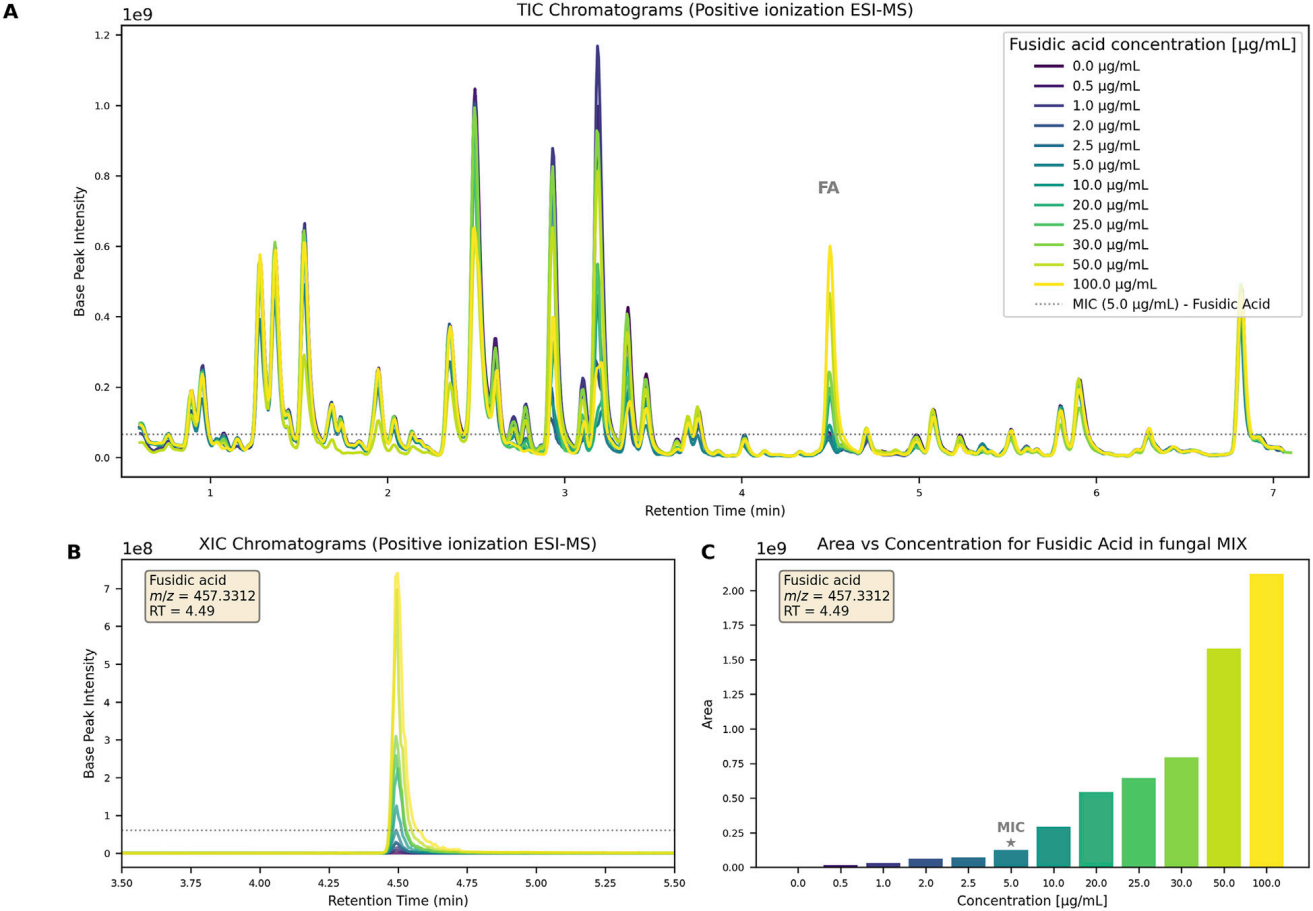
**(A)** TIC chromatograms of the complex fungal extract at 5 mg/mL spiked with increasing FA concentration (0-100 μ g/mL). The peak corresponding to FA is annotated with FA in grey. All extracts were tested against the pathogen *Staphylococcus aureus* and the dotted line represents the intensity of signal corresponding to MIC value. **(B)** XIC chromatogram of the mass: *m/z* = 457.3312, RT = 4.49 min across all spiked extracts. The dotted line represents the intensity of signal corresponding to MIC value. **(C)** Area of the FA feature obtained in positive ionization mode plotted against the concentration in fungal complexe mixture. The MIC value is labeled with a star.

To establish detection limits, a biologically inactive extract was prepared by pooling five fungal cultures grown in different media, extracted, and re-suspended in MeOH (5 mg/mL). UHPLC-HRMS/MS analysis confirmed its chemical richness (Figure 3A, 0.0 μ g/mL). This extract was then spiked with fusidic acid (FA), a known antimicrobial agent of fungal origin (Godtfredsen et al., 1962; Verbist, 1990).

To mimic conditions obtained after small-scale extraction, 0.5 mg of extract was spiked with increasing amount of FA (0-5 μ g) and re-suspended in 100 μ L of DMSO. As a control, identical dilutions were prepared in DMSO without the fungal extract. The Minimum Inhibitory Concentration (MIC) against *S. aureus* was identical in both the spiked fungal extract and the DMSO control solutions, demonstrating the bioassay’s compatibility with complex mixtures. This confirms that no matrix effects or inhibitory interferences were observed under the tested conditions. The bioassay detection threshold was determined to be 5 ppm (0.5 μ g of FA spiked in 0.5 mg of extract, re-suspended in 100 μ L of DMSO).

Similarly, to assess MS detection thresholds, all samples were analyzed using the generic non-targeted UHPLC-HRMS/MS method (see 5.9.3). FA was monitored via extracted ion chromatograms (XIC) of its [FA-acetyl-H_2_O]^+^ main ion (Figure 3B). It was detectable even in the lowest concentration sample (0.5 ppm). The feature intensity was plotted against concentration, demonstrating that MS detection was sufficiently sensitive, with a detection limit at least 10 times lower than the MIC (Figure 3C).

In the 5 ppm spiked extracts, FA was readily detected, with signals intensities reaching 1E08 (Figure 3A, dotted line indicating FA intensity at MIC). This signal intensity level ensured that high-quality ${\text{MS}}^{2}$ spectra could be obtained for such bioactive compound in extract for further dereplication and annotation in our metabolomic workflow.

These results indicate that our platform can reliably detect as little as 0.5 μ g/well (5 ppm) of an active ingredient by MS and obtain a hit in bioassay, provided the active ingredient exhibits similar bioactivity and ionization properties to fusidic acid.

### 2.2 Application of the developed workflow for hit identification

#### 2.2.1 Combination of metabolomics and bioassay results of fungal strains replicates

The plate previously used for metabolomics profiling (ABO-P26) was subjected to antimicrobial screening assays against the pathogen *Staphylococcus aureus*. Nine extracts showed growth inhibition comparable to vancomycin control, with a few additional samples at the threshold (within one SD above the positive control). Six of these active extracts originated from six independent replicates of a single *Chaetomium globosum* strain, highlighting the robustness of the protocol. In contrast, another *C. globosum* strain, also tested in six replicates, showed no detectable bioactivity, suggesting strain-specific differences in metabolite production. Metabolomics was used to investigate the chemical basis of these differences and to identify potential bioactive candidates.

To confirm that the two *C. globosum* strains were different, an additional PCA was performed on this subset. This revealed two distinct groups based on their strain origin (see right panels of Figure 2) with PC1 and PC2 explaining almost 80% of the dataset variance.

MS features detected in *C. globosum* were categorized into three groups: exclusive to the active strain (143 features), exclusive to the non-active strain (442 features), and shared between both (338 features). Features were filtered to retain those detected in at least 4 out of 6 replicates, and average intensities were calculated. From these, we focused on 276 features of interest: 143 exclusive to the active strain and 133 shared but more abundant in active ones (fold change ≥ 1).

**FIGURE 4:**
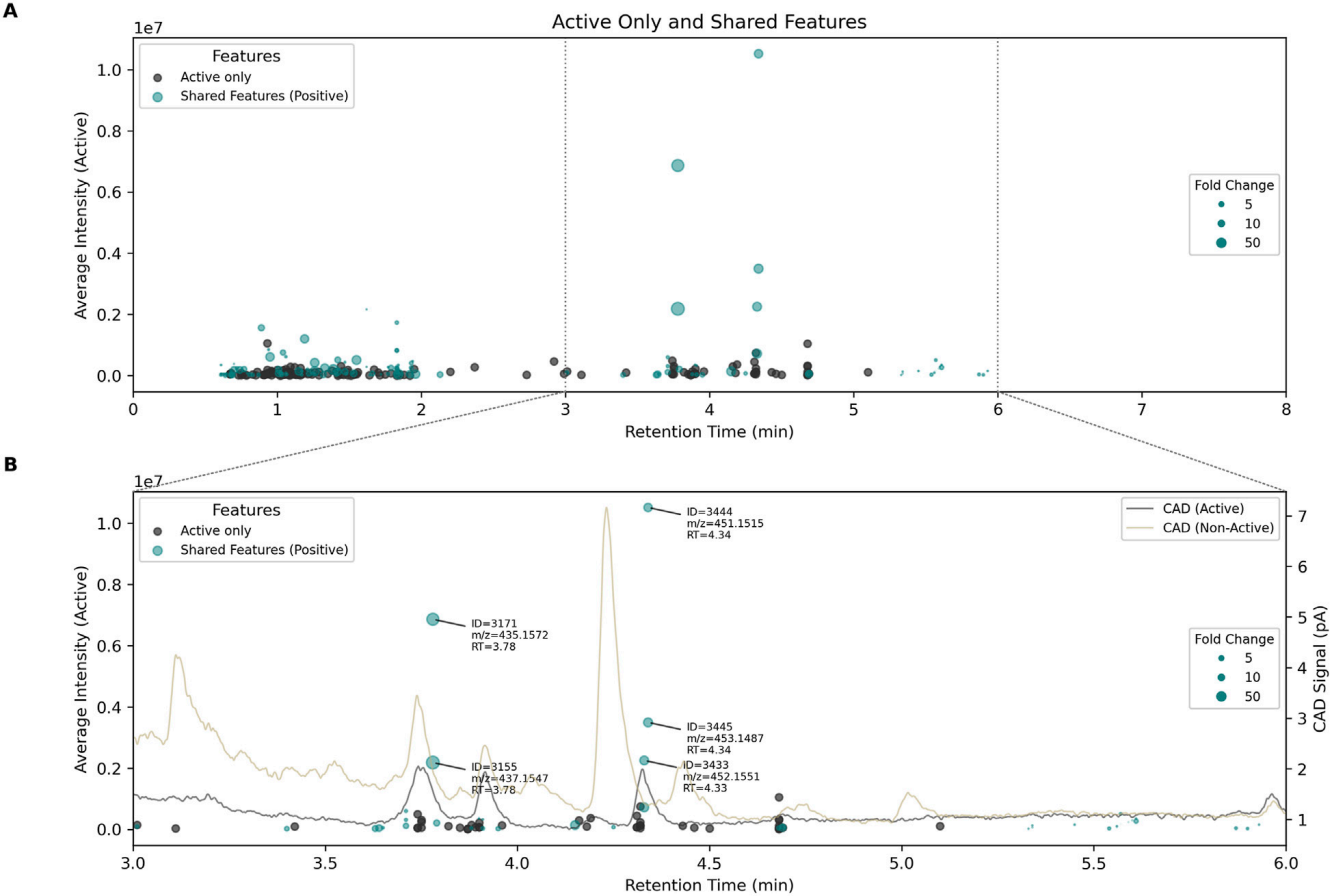
**(A)** chromatogram like plot showing features exclusively present in active *Chaetomium globosum* extracts (dark dots) as well as shared features (blue dots). Features are displayed according to their retention times (x-axis) and mean intensity (y-axis). The size of the shared features corresponds to the fold change ( ≥ 1). The dotted lines at 3 and 6 min represent the zoomed region of plot **(B)** Focus between 3 and 6 min. The CAD traces of representative active (dark grey line) and non-active samples (beige line), retention times (RT) feature ID’s and mass to charge ratios (*m/z*) are also shown.

To potentially identify the LC peak(s) responsible for bioactivity, these 276 features were visualized in a chromatogram-like plot (Figure 4A), with retention time on the x-axis and average intensity on the y-axis. Most features exclusive to active samples (dark dots) had low MS intensities ( ≤ 2E6), suggesting they were unlikely contributing to bioactivity. For shared features (blue dots), dot size was proportional to the calculated fold change, highlighting several metabolites significantly more abundant in active samples. Interestingly, these features also exhibited high intensity and eluted between 3 and 6 min.

To complement MS data, semi-quantitative traces were simultaneously acquired using a Charged Aerosol Detector (CAD), which provides signal response independently of compound type (Vehovec and Obreza, 2010). Representative chromatograms from active and non-active *C. globosum* groups are shown in Figure 4B, which provides a zoomed view of shared features (blue dots) with high intensity (high y-values) and significant fold changes (large dots). The most abundant features identified earlier correlated with relatively intense CAD signals, confirming that they represent major constituents of the extracts. Notably, five features (ID = 3171, 3155, 3444, 3445, 3433) were observed at two retention times (3.78 min and 4.34 min), suggesting the presence of two distinct molecules. In both cases, isotopic patterns indicated the presence of a chlorine atom, with mass differences between features supporting this hypothesis. These molecules were also associated with distinct CAD peaks, reinforcing their status as major components and strong bioactive candidates. This correlation between bioactivity, CAD intensity, and MS features is valuable for linking extract-level activity to specific compounds and supports the feasibility of isolating sufficient quantities for further characterization.

**FIGURE 5:**
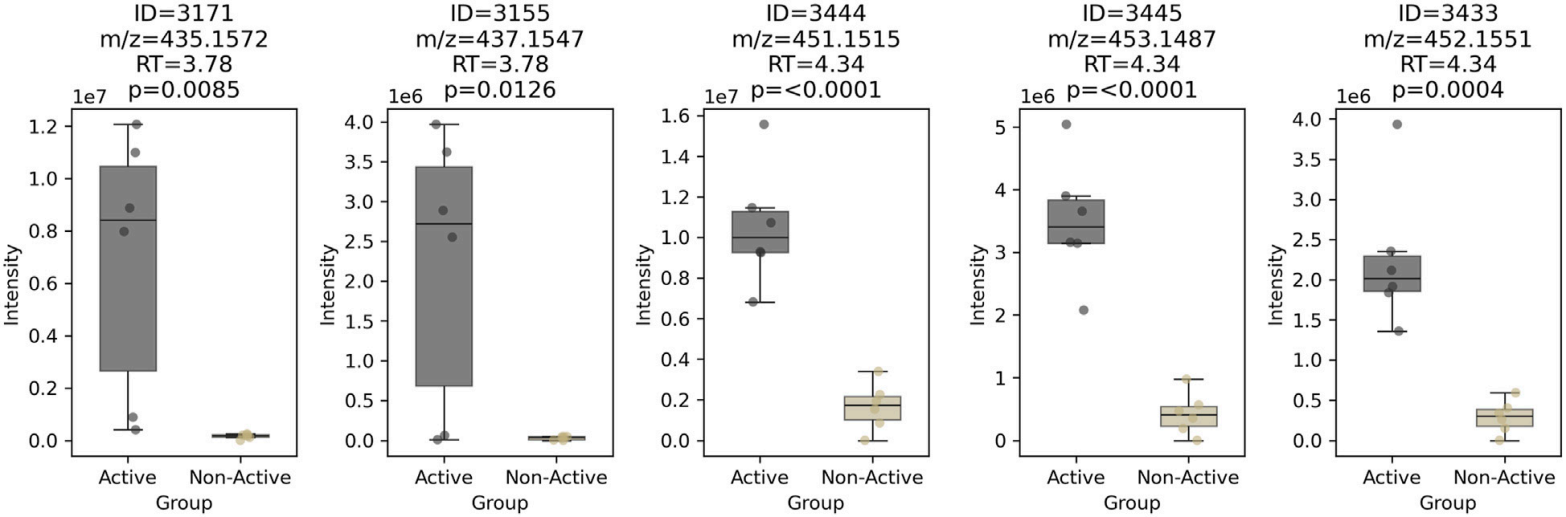
Box-plot of the 5 most abundant features shared between active and non-active *Chaetomium globosum* samples (n = 6). The corresponding feature ID, *m/z*, retention time and p-value after one-way ANOVA are displayed on top of each box-plot.

A box plot of these five features clearly distinguished the two strains (active vs. non-active in Figure 5). Features at RT = 3.78 min (ID = 3171 and 3155) appeared in only 4 out of 6 active samples, making the corresponding compound a less likely candidate for bioactivity. Conversely, three features at RT = 4.34 min (ID = 3444, 3445, and 3433), were significantly more abundant in all active samples (n = 6/6), increasing the likelihood that this chlorinated molecule was the bioactive candidate.

Annotation using SIRIUS (Dührkop et al., 2019) proposed this compound as belonging to the chaetoviridin or chaetomugilin classes of azaphilones. The widespread occurrence and bioactivities of azaphilones in various *Chaetomium* species, particularly *C. globosum*, have been extensively described (Pavesi et al., 2021). Notably, several azaphilones have been reported to exhibit antimicrobial activity (Wang et al., 2018; Shen et al., 2023). This putative bioactive chlorinated azaphilone presented a molecular formula of ${\text{C}}_{23}{\text{H}}_{27}{\text{ClO}}_{7}$ and an exact mass of 450.1445. Features 3444 and 3445 corresponded to [M + H]^+^ and [M + H+2]^+^, the two isotopes of chlorine, while feature 3433 ([M + H+1]^+^) likely represented its ^13^C isotope.

##### 2.2.1.1 HPLC-based antimicrobial activity profiling of *Chaetomium globosum*

**FIGURE 6:**
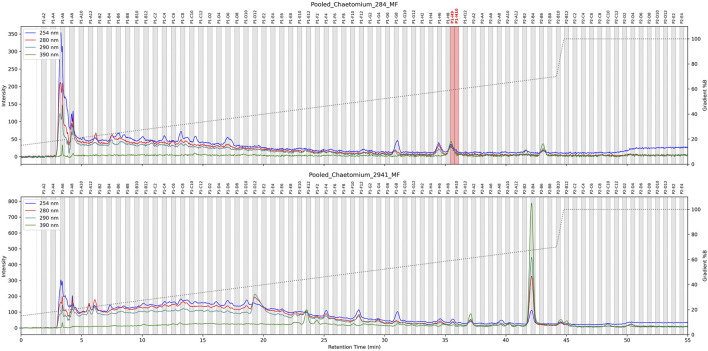
HPLC-based antimicrobial activity profiling of two *C. globosum* strains cultured in replicate (n = 6). For each strain, the six extracts were combined and subjected to HPLC-UV fractionation under an optimized gradient. The resulting microfractions were collected and tested for bioactivity. Active fractions against *Staphylococcus aureus* are highlighted in red.

To confirm these findings, the six extracts of each *C. globosum* were combined and dried, yielding 2.3 and 3.4 mg for the active and non-active strains respectively. These extracts were then subjected to high resolution HPLC-based micro-fractionation under optimized gradient conditions, resulting in 165 fractions of 1.5 mL for each strains. All fractions were tested for their bioactivity and, as expected, only one LC-peak (spread in two fractions) from the active *C. globosum* strain exhibited activity against the pathogen *Staphylococcus aureus* (see Figure 6).

UHPLC-HRMS/MS analysis confirmed that active fractions contained a compound matching previously identified features IDs 3444 and 3445. Examination of SIRIUS annotations, the molecular formula ( ${\text{C}}_{23}{\text{H}}_{27}{\text{ClO}}_{7}$ ), ${\text{MS}}^{2}$ fragmentation patterns, and chemotaxonomic data putatively identified 12- β -Hydroxychaetoviridin C as the bioactive metabolite—a known compound for which anti-*Staphylococcus aureus* activity had not been previously reported (Borges et al., 2011).

#### 2.2.2 Identification of bioactive scaffolds using molecular networking in a single-replicate fungal strain collection

After demonstrating high specificity and reproducibility with replicate samples (n = 6) across 14 strains, we applied our screening approach to a set of 90 fungal strains (n = 1 each), selected to ensure broad taxonomic coverage. These strains (detailed list in Supplementary Material) were cultivated in a single 96-well plate, including six media-only controls (PDB). After two weeks of incubation, extracts were subjected to metabolomic profilling, and tested for antimicrobial activity against *S. aureus*. Among the 90 extracts, only one, from *Verticillium lateritium*, showed antimicrobial activity.

**FIGURE 7:**
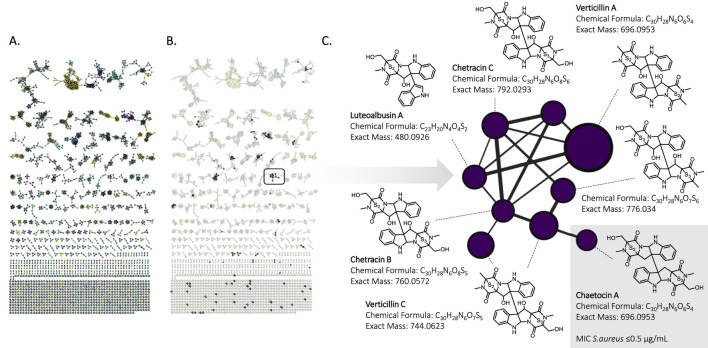
**(A)** Feature-Based Molecular Network of 90 fungal strains extracts obtained from positive ionization mode high-resolution tandem mass spectrometry. Each color corresponds to a specific strain. **(B)**. Extracted features of the active extract. **(C)**. Bioactive Epipolythiodioxopiperazines cluster with its corresponding annotations performed with SIRIUS. Chaetocin A was active against *Staphylococcus aureus* (MIC ≤ 0.5 μ g/mL). The size of the nodes is proportional to the ${\text{MS}}^{1}$ intensity of the feature.

To identify the potential bioactive compound(s), a FBMN was constructed using ${\text{HRMS}}^{2}$ data. It displayed 2,738 features across all extracts and provided a comprehensive overview of the metabolites’ chemical space (Figure 7A). To identify key bioactive compounds within this large dataset, features unique to the active strain were highlighted in the FBMN, revealing individual nodes and clusters (Figure 7B). In particular, a distinct cluster of high-molecular weight features ( ≈ 700 Da) only exclusive to *V. lateritium* was identified. It was consistently annotated by SIRIUS as epipolythiodioxopiperazine (ETP) alkaloids across the entire cluster (Figure 7C). These fungal secondary metabolites, often characterized by a dimeric structure, contain a polysulfur bridge on the dioxopiperazine moiety.

One node of this cluster was annotated as chaetocin A. To confirm its identity, a standard was purchased and was analyzed by LC-HRMS/MS in the same condition. It exhibited a hight ${\text{MS}}^{2}$ spectral similarity and matched the retention time. Additionally, it showed potent anti-*S. aureus* activity (MIC ≤ 0.5 μ g/mL), thus clearly confirming that it was one of the active ingredients.

The confirmation of the structure and activity using a commercial standard of chaetocin A demonstrates the robustness of the annotation and the efficiency of the methodology to rapidly pinpoint active ingredients in a complex mixture.

## 3 Discussion

In our study, we demonstrated that small-scale fungal liquid culture is a valid alternative to traditional fermentation methods, which often require longer incubation periods and large vessel containers. This approach offers valuable advantages for comparative studies by enabling the parallel and compact cultivation of numerous fungal strains, ensuring high consistency and throughput. Moreover, the small-scale format is highly adaptable to various liquid media, further enhancing its versatility.

Solid-phase extraction (SPE) (Chen et al., 2019) was identified as the preferred method for rapidly generating extracts (5 mg/mL in DMSO) directly in 96-well plate formats due to its straightforward sample preparation, superior concentration factor, widespread commercial availability, remarkable recovery rates and reduced variability (see Figure 1). This method enabled the processing of nearly 200 samples (2 plates) in under 2 hours following growth, including mycelium disruption, centrifugation, and SPE enrichment, thereby significantly enhancing productivity while maintaining high-quality results.

Despite the limited scale, extract yields on the mother plate (approx. 100 μ L at 5 mg/mL per well) were sufficient to carry out a first representative antimicrobial bioassay and MS-based metabolomic analyses. Moreover, the available quantity is likely suitable for at least 5 to 10 additional bioassays, enabling a more comprehensive characterization of the extracts’ bioactivity profiles.

A rapid chemical exploration of the extracts was achieved through systematic non-targeted metabolomics profiling Defossez et al. (2023). This approach, which was also used to comprehensively monitor various extraction protocols, provided valuable insight into each strain’s secondary metabolite profile. It allowed us to evaluate the impact of culture conditions and scale on metabolite production, detect strain-specific compounds, and thus confirm the absence of cross-contamination in deep-well plate formats.

This strategy proved particularly useful for comparing fungal strains on the basis of their metabolic profiles, enabling approaches like multivariate data analysis (Selegato et al., 2022) and Feature-Based Molecular Networking (FBMN) (Nothias et al., 2020). The latter aligns with current trends in fungal research (Fan et al., 2022). In addition, we demonstrated that the developed workflow effectively documented both the biological activity and chemical composition of each strain. Combining these results with appropriate data processing enabled to identify features potentially linked to the observed biological activities at the extract level.

Applying this workflow to the cultivation and analysis of replicated strains of the same fungal species, enabled the statistical evaluation of features that were strain-specific or significantly overexpressed in active extracts compared to non-active ones. Furthermore, the integration of a universal detection method (CAD) (Vehovec and Obreza, 2010) into the analytical platform provided semi-quantitative information on these highlighted features based on their retention times. When combined with the structural insights offered by HRMS data, this complementary approach was key to hypothesizing the nature of the bioactive compounds within the extract. The availability of six replicates per strain allowed for the pooling of samples for HPLC bioactivity profiling (Hamburger, 2019), enabling the evaluation of antimicrobial activity across the entire chromatogram (see 6). The results confirmed that metabolomic features highlighted by statistical analysis corresponded to the observed chromatographic bioactive region.

For plate-based screening of fungal strains, the approach proved effective even with a single replicate per strain. Massive Multi-informational Molecular Networks (Olivon et al., 2017) enabled the exploration of 90 fungal strains producing nearly 3000 features and the effective identification of an active metabolite among this large dataset. While not always straightforward, the process could benefit from recent advances in the field such as integrating advanced statistical methods (Pakkir Shah et al., 2025) or NMR information Meunier et al. (2024) within molecular networks.

Thus, the presented approach has demonstrated its potential for large-scale fungal strain screening. By integrating MS data within a carefully designed experimental framework, it enables the selection of strains of interest through the dereplication of known compounds while advanced statistical approaches (Issaq et al., 2009; Rinschen et al., 2019) could strengthen our workflow by refining the identification of key bioactive compounds. The large volume of data generated by our platform is particularly valuable in this context and could accelerate the discovery of novel bioactive molecules, especially when working with extensive fungal strain libraries that would otherwise be challenging to exploit conventionally.

Ultimately, the platform is designed to be adaptable: researchers can adjust data filtering parameters based on their objectives. When sensitivity is prioritized over throughput, the abundance threshold can be lowered to retain more features for follow-up. Once a bioactive signal is observed, further investigations can be carried out to prevent the rediscovery of known molecules and to uncover bioactive minor metabolites.

Despite its clear advantages, our approach does have certain limitations. Some highly sporulating fungal species can pose challenges when cultivated in deep-well plates, requiring meticulous care during inoculation to avoid issues such as cross-contamination. For example, contamination with *Penicillium expansum* strains has been observed (data not shown). This contamination problem is particularly well documented in bacterial fermentation using similar plate formats but seams to occur rather during the extraction steps than the culture itself (Minich et al., 2019; Walker, 2019). Furthermore fungal species exhibit diverse phenotypes, including various growth rates Meletiadis et al. (2001) and sporulation (Dijksterhuis, 2019). To ensure uniform biomass development in deep-well plates and minimize experimental variability (extract yields), it is advisable to group species with similar growth rates.

The SPE extraction process was optimized for compounds with physicochemical properties similar to known antibiotics. To expand the range of extracted metabolites, the protocol can be refined by adjusting the polarity or pH of the elution solvent for further enrichment (Chen et al., 2019).

The presented approach, integrating systematic metabolomic profiling, was designed to efficiently screen a large number of fungal strains. Although the small-scale format does not yield sufficient material for full *de novo* structure elucidation, it does enable the rapid identification of active features. Once an active compound is identified, maintaining the plate-based culture format allows for parallel testing of different culture conditions—for instance, through OSMAC approaches Bode et al. (2002); Daletos et al. (2017)—to increase the target compound’s titer (Michaliski et al., 2023). Systematic MS-based profiling further enables the monitoring of features of interest, thereby leveraging the multiplexing capabilities of the developed methodology. This can significantly accelerate the optimization process while reducing reliance on bioassays.

Once optimal conditions are established, the culture can be scaled up ( ≤ 100 mL) to produce enough material for comprehensive characterization of the active metabolites. At this stage, the informed use of MS data becomes instrumental for the targeted isolation of the bioactive compound. However, scaling up is not without challenges. Due to the metabolic plasticity of fungi, metabolite production may vary with changes in culture scale, even under otherwise identical conditions (Ganeshan et al., 2021). Nevertheless, we have demonstrated that the cultures are reproducible between wells and that pooling replicates yields consistent results. Scaling up across an entire plate is therefore advisable, as it can generate extract quantities in the range of tens of milligrams—sufficient for comprehensive characterization, including NMR quantification, and for bioassays requiring only a few tens of micrograms of purified product.

## 4 Conclusion

The FLECS-96 approach combining small-scale fungal strain cultivation and extraction has demonstrated its effectiveness and robustness in producing metabolite enriched extracts for rapid screening of hundreds of strains. Our sample preparation method exhibited high reproducibility and compatibility with MS based metabolomics workflows, enabling quick hypothesis generation regarding the identity of active compounds. This supports informed decisions on scaling up strain production or directly testing pure compounds when available.

By integrating streamlined extract generation with sensitive antimicrobial bioassays, advanced metabolomics workflows and statistical approaches, the methodology facilitates mass-guided optimization of cultivation conditions. This enables efficient scale-up under optimized conditions and targeted isolation of novel bioactive NPs, significantly reducing the workload and costs associated with compound isolation.

In summary, our methodology provides a highly efficient process for the discovery and characterization of potential antimicrobial agents from fungal sources, effectively transforming strain collections into workable extracts with documented composition, thereby accelerating the early stages of drug discovery. Its implementation as a screening and prioritization tool could reinvigorate antimicrobial discovery campaigns from fungal strains. Moreover, this approach, while exemplified in antimicrobial drug discovery, is readily adaptable to other bioassays, offering broader applications in drug discovery across diverse therapeutic areas.

## 5 Materials and Methods

### 5.1 Chemicals

Unless otherwise specified, all chemicals were purchased from Sigma-Aldricht.

### 5.2 Fungal strains collection

The fungal strains used in this study are part of Agroscope’s “Mycotheca” collection, stored at Changins, Switzerland, and are publicly accessible online (https://www.mycoscope.ch). This collection includes various fungal strains maintained for research purposes, stored on minimal culture media in refrigerators at 4°C. Each strain is assigned a unique ID and accompanied by detailed taxonomic information.

### 5.3 DNA extraction and PCR Amplification

A 100 mg sample of fungal culture from PDA plates was transferred into 550 μ L of CD1 buffer (DNeasy Plant Pro Kit, ref. 69204). Samples were homogenized using a TissueLyser II (Qiagen, ref. 1218130627E) at 24 Hz for 3 min. The lysate was transferred to a 2 mL collection tube and subjected to DNA extraction using a QIAcube system (QIAcube Connect Priority Blue 279, ref. 9002842), with DNA eluted in 100 μ L of EB buffer. PCR was performed using 5 μ L of extracted DNA in a reaction mixture containing 2.5 μ L of 10 × Buffer, 2.25 μ L of 25 mM ${\text{MgCl}}_{2}$ , 1.25 μ L each of ITS4 and ITS1F primers, 1 μ L of dNTPs, 1 μ L of betaine, 0.125 μ L of Taq DNA polymerase, and 10.625 μ L of Milli-Q water. The PCR conditions included an initial denaturation at 94°C for 2 min, followed by 30 cycles of 94°C for 30 s, 55°C for 30 s, and 72°C for 30 s, with a final extension at 72°C for 10 min. PCR products were visualized on a 1.8% agarose gel. Sequences were analyzed via BLAST^®^ against GenBank for strain identification.

Strain identification was based on the PCR products amplified using ITS4 (TCC TCC GCT TAT TGA TAT GC) and ITS1F (CTT GGT CAT TTA GAG GAA GTA A) primers. Sequencing was performed by Genesupport SA (Fasteris Sanger Services, CH-1228 Plan-les-Ouates). The resulting sequences were compared against the GenBank BLAST^®^ database (https://blast.ncbi.nlm.nih.gov/Blast.cgi) to identify species.

### 5.4 Culture media preparation

Potato Dextrose Agar (PDA) was prepared by dissolving 39 g of PDA powder (Potato Dextrose Agar, NutriSelect Plus (Dr. Grogg, 70139-2.5 KG)) in 1 L of Milli-Q water, autoclaving for 30 min at 121°C, and pouring into sterile 9 cm or 14 cm Petri dishes, depending on usage.

Potato Dextrose Broth (PDB) was prepared by dissolving 24 g of PDB powder (Potato Dextrose Broth, NutriSelect Basic (Dr. Grogg, P6685-1 KG)) in 1 L of Milli-Q water, distributing 100 mL into 250 mL Erlenmeyer flasks, sealing with cellulose plugs and aluminum foil, and autoclaving for 30 min at 121°C.

*Botrytis cinerea* Media (BC) was prepared by dissolving the following components in 1 L of warm Milli-Q water: 1g Glucose, 5g anhydrous ${\text{KH}}_{2}{\text{PO}}_{4}$ , 2.5g ${\text{MgSO}}_{4}\cdot 7{\text{H}}_{2}\text{O}$ , 10g ${\text{NaNO}}_{3}$ , 1g ${\text{CaCl}}_{2}\cdot 2{\text{H}}_{2}\text{O}$ , 1mg ${\text{FeCl}}_{3}$ , 15g Pectin, and 1mg Thiamine. Sterilize the medium by autoclaving at 121°C for 30 min. Allow the medium to cool to room temperature before use.

Lysogeny Broth (LB) was prepared by dissolving 25 g of LB powder in 1 L of Milli-Q water. Dissolve completely and autoclave at 121°C for 30 min. Allow the medium to cool to room temperature before use.

Antimony-enriched Potato Dextrose Broth (PAT) was prepared by dissolving 5 g of powdered Potassium antimony tartrate ${\text{C}}_{8}{\text{H}}_{4}{\text{K}}_{2}{\text{O}}_{12}{\text{Sb}}_{2}\cdot 3{\text{H}}_{2}\text{O}$ (Sb) and 24 g of PDB powder (Potato Dextrose Broth, NutriSelect Basic (Dr. Grogg, P6685-1 KG)) in 1 L of Milli-Q water. Dissolve completely and autoclave at 121°C for 30 min. Allow the medium to cool to room temperature before use.

Copper-enriched Potato Dextrose Broth (Cu) was prepared by dissolving 24 g of PDB powder (Potato Dextrose Broth, NutriSelect Basic (Dr. Grogg, P6685-1 KG)) and 5 mg ${\text{CuSO}}_{4}$ in 1 L of Milli-Q water. Dissolve completely and autoclave at 121°C for 30 min. Allow the medium to cool to room temperature before use.

### 5.5 Fungal strain reconditioning

Under sterile conditions, fungal strains were reconditioned from the Mycotheca. The tube containing the fungal strain was opened, and a piece of mycelium was transferred to a PDA Petri dish using a sterile lancet. Plates were sealed with Parafilm and incubated for approximately 2 weeks, or until full mycelial coverage. For subculturing, a 5 mm × 5 mm square of mycelium was excised and transferred to a fresh PDA plate, ensuring direct contact with the medium.

### 5.6 96-Well plate preparation and inoculation

A deep-well 96-well plate (104099, Kuhner Shaker) was prepared with sterile PDB (24 g/L) by pipetting 1.8 mL into each well. The plate was sealed with a Cap-mat (186006335, Waters) and a lid (104106, Kuhner Shaker) before autoclaving at 121°C for 30 min. In a sterile environment, a 2 mm × 2 mm mycelial piece was excised with a sterile lancet and carefully placed in the designated well, avoiding spore dispersion. The plate was sealed and tightly clamped using the Duetz system (Duetz, 2007) (see also https://enzyscreen.com), then incubated for approximately 14 days at room temperature under a 12-h light/dark photoperiod.

### 5.7 Small-scale extraction protocols exploration

#### 5.7.1 96-Well plate extraction protocol

After the growing period, a 3 mm Tungsten Carbide Bead (3 mm, Qiagen^®^, Cat. No. 69997) was added to each well. The plate was sealed with a Cap-mat (186006335, Waters) and a lid (104106, Kuhner Shaker) before being placed in the TissueLyser II (Qiagen^®^ Hilden, Germany) for 5 min of homogenization at 30 Hz. The plate was then centrifuged for 1 hour at 4,300 rpm (sorvall^®^ super T21).

Simultaneously, C18-SPE cartridges in 96-well format (Discovery DSC supelco^®^ Merck, Darmstadt, Germany) were conditioned with 1 mL MeOH (HPLC grade, Fisher Scientific) followed by 1 mL of water. Elution was performed using a vacuum manifold adapted for 96-well plate format (supelco^®^ Merck, visiprep Darmstadt, Germany).

Following centrifugation, 1 mL of the resulting supernatant was loaded onto the conditioned C18-SPE cartridges and washed twice with 1 mL of water. Extracts were eluted with 1 mL MeOH (HPLC grade, Fisher Scientific) and collected in pre-weighted individual wells (1.2 mL capacity) of a 96-well plate (Costar^®^, Corning, United States).

The plate of extract was concentrated with a Genevac^®^ EZ-2 Elite (SP Industries, Warminster, Pennsylvania) for 10 h. Samples were then manually weighed, and an appropriate volume of DMSO (LC-MS grade, Thermo Scientific, REF 85190) was added to each well to adjust the final concentration of extract to 5 mg/mL.

#### 5.7.2 QuEChERS extraction

Original QuEChERS protocols were sourced in literature (He et al., 2018; Zhu et al., 2021; Brosnan et al., 2014; Desmarchelier et al., 2018) and adapted accordingly to our samples. The QuEChERS method involves extraction with a solvent, followed by induced liquid–liquid partitioning through the addition of a buffer or non-buffered salt. A dispersive solid-phase extraction (d-SPE) is then performed to clean up the extracts and reduce matrix interferences. To miniaturize the extraction for use in 96-well plates, this two-step protocol required two separate plates: one for each step.

Given the well capacity of a 96-deepwell plate (2 mL), protocols were adapted to accommodate an arbitrary supernatant volume of 1 mL (see Supplementary Figure S2). The solvent volume was fixed at 750 μ L, resulting in a total volume of 1.75 mL per well. These volumes were added to the first QuEChERS plate containing salts necessary for liquid–liquid partitioning. To ensure proper extraction, a 3 mm Tungsten Carbide Bead (Qiagen^®^, Cat. No. 69997) was added to each well. The plate was sealed with a Cap-mat (Waters, 186006335) and a lid (Kuhner Shaker, 104106), homogenized in a TissueLyser II (Qiagen^®^, Hilden, Germany) at 30 Hz for 2 min, and centrifuged at 4,000 rpm (sorvall^®^ super T21) for 15 min.

In the second step, 650 μ L of the upper organic phase from the first step was transferred into a 96-well plate containing different d-SPE combinations. A 3 mm Tungsten Carbide Bead (Qiagen^®^, Cat. No. 69997) was added to each well and the mixture was shaken 1 minute at 30 Hz using the TissueLyser II and centrifuged at 4,000 rpm for 10 min. Then, 600 μ L of supernatant was collected, filtered through a 0.2 μ m AcroPrep 96 filter plate (PALL Life Sciences, PN 5052) and evaporated to dryness in a Genevac at 40°C. Finally, the residue was reconstituted with 100 μ L of a mixture of acetonitrile and water (0.1% formic acid), 5:95 (v/v) and transferred into an autosampler vial for UHPLC-HRMS/MS analysis (see Supplementary Figure S2).

For both steps of each protocol, mixtures corresponding to 25 wells were prepared in bulk in a flask, homogenized, and aliquoted into individual wells. The detailed preparation of these mixtures is given in Supplementary Material.

#### 5.7.3 Supported liquid extraction (SLE)

Supported Liquid Extraction (SLE) cartridges were loaded with 500 μ L of spiked samples under light vacuum, allowing the samples to slowly permeate the cartridges. After a 5-min equilibration period, elution was performed using four 500 μ L aliquots of ethyl acetate (EtOAc). The combined eluates were then concentrated to dryness *in vacuo* using a Genevac at 40°C. Finally, the residue was reconstituted with 100 μ L of a mixture of acetonitrile and water (5:95, v/v) containing 0.1% formic acid, and transferred into an autosampler vial for UHPLC-HRMS/MS analysis.

### 5.8 Recovery rates and coefficient of variation calculation

#### 5.8.1 Individual stock solutions (2 mg/mL)

An individual stock solution (2 mg/mL) was prepared by dissolving 2 mg of each antibiotic listed in Table 1 in 1 mL of DMSO (LC-MS grade, Thermo Scientific, REF 85190).

#### 5.8.2 Mix stock solution (100ppm)

From these eleven individual stock solutions, a standard mix solution at 100 μ g/mL (100 ppm) concentration was prepared by transferring 100 μ L of each solution into a 4 mL vial. ${\text{H}}_{2}\text{O}$ /ACN (90/10; 900 μ L) was added to this vial to bring the total volume to 2 mL.

#### 5.8.3 Final mix solutions (0–100ppm)

Calibration curves solutions (S0–S7) at concentrations of 2.5 ppm, 5 ppm, 10 ppm, 25 ppm, 50 ppm, 75 ppm and 100 ppm were prepared weekly by accurately pipetting volumes of 25 μ L, 50 μ L, 100 μ L, 250 μ L, 500 μ L, 750 μ L and 1000 μ L respectively from the standard mix solution (100 ppm) into an amber vial (1.5 mL) and each vial was made up to a total volume of 1 mL with DMSO (see Table 2).

**TABLE 2:** Concentration obtained after dilution of the mix stock solution at 100 ppm with DMSO. Each solutions was prepare 3 times per level in order to have 3 points per concentration for calibration.

Levels	Concentration [ppm]	VOL. SOL. Stock MIX	VOL DMSO
S0	0.0	0 μ L	1000 μ L
S1	2.5	25 μ L	975 μ L
S2	5.0	50 μ L	950 μ L
S3	10	100 μ L	900 μ L
S4	25	250 μ L	750 μ L
S5	50	500 μ L	500 μ L
S6	75	750 μ L	250 μ L
S7	100	1000 μ L	0 μ L

Freshly prepared solutions (S0-S7) were injected into the UHPLC-HRMS/MS for calibration in analytical triplicates (A,B,C) using our generic chromatographic method described in section 5.9.3.

##### 5.8.3.1 Calibration curves

Calibration curves were constructed by serial dilutions of individual stock solutions for each antibiotic as described above. Each solution was injected in our generic reverse phase UHPLC-HRMS/MS chromatographic method described in chapter 4.9.3. Retention times (RT) for each antibiotics were determined and an integration window of RT +/- 0.5 min was used in combination with the *m/z* value to detect and integrate each analytes using Skyline software (version 23.1 (64-bit)). Integrated area were put in line with their calculated concentration (from 0 to 100 ppm) to construct individual calibration curves.

Calibration curves were constructed using serial dilutions of individual stock solutions for each antibiotic, as described previously. These diluted solutions were analyzed using our generic reverse-phase UHPLC-HRMS/MS chromatographic method (detailed in Section 5.9.3).

For each antibiotic:

1. Retention times (RT) were determined.
2. An integration window of RT ± 0.5 min was established.
3. This window was used in conjunction with the specific *m/z* value to detect and integrate each analyte.

##### 5.8.3.2 Spiked matrices

Three different matrices were spiked with the eleven antibiotics (at 5 ppm each) and used to evaluate the different extraction protocols. The three selected matrices are listed here:

• Milli-Q water
• PDB (prepared as described above)
• A fungal broth filtrate

##### 5.8.3.3 Spiked antibiotics on a fungal broth

The fungal broth used was the filtrate of a 2-L *Penicillium expansum* (Mycotheca ID: 1847) culture. After a 2-week period, the culture broth was filtered on 0.22 μ m and used directly in the spiking experiments.

##### 5.8.3.4 Spiked fusidic acid on a complexe fungal mixture

Five fungal strains were cultured separately: *Botrytis cinerea* (2992), *Epicoccum nigrum* (10), *Exophiala oligosperma* (2636), *Laetisaria fuciformis* (1691), and *Botryosphaeria obtusa* (1026). In order to express as much metabolites as possible out of the five strains, we decided to culture them on five different media. Each strain was grown in 50 mL Falcon tubes containing 25 mL of different media: PDB, PAT, LB, Cu, and BC (media compositions described above). This resulted in 25 individual cultures (5 strains × 5 media).

This step was designed to enhance the chemical diversity of the fungal cultures, thereby producing a richer extract for method development and validation.

The cultures were filtered and pooled, yielding 625 mL of combined culture supernatant. This pooled supernatant underwent liquid-liquid extraction with ethyl acetate (EtOAc, 3 × 250 mL). The organic phases were washed with water (3 × 250 mL) and concentrated *in vacuo* at 40°C to obtain a crude extract.

The crude extract was resolubilized in a mixture of MeOH/water (7:3) and defatted by partitioning with hexane (3 × 250 mL). This process yielded 250 mg of ABO-QC-MIX. ABO-QC-MIX was then re-solubilized in MeOH at a concentration of 5 mg/mL and injected in UHPLC-HRMS/MS to control its expected metabolite richness. Having shown a diversified profile, Fusidic acid was then spiked at different concentrations into the ABO-QC-MIX.

### 5.9 Chromatographic separations

#### 5.9.1 UHPLC-PDA-ELSD-MS analysis

Analysis were performed on an Ultra-High-Performance Liquid Chromatography system equipped with a PhotoDiode Array, an Evaporative Light-Scattering Detector, and a single quadrupole Mass Spectrometer detector using heated electrospray ionization (UHPLC-PDA-ELSD-MS) (Waters, Milford, MA, United States).

The ESI parameters were as follows:

• Capillary voltage: 800 V
• Cone voltage: 15 V
• Source temperature: 120°C
• Probe temperature: 600°C

Acquisition was conducted in positive or negative ionization mode with an *m/z* range of 150–1000 Da. Chromatographic separation was performed on an Acquity UPLC BEH C18 column (50 × 2.1 mm i.d., 1.7 μ m; Waters, Milford, MA, United States) at 0.6 mL/min, 40°C with ${\text{H}}_{2}\text{O}$ (A) and ${\text{CH}}_{3}\text{CN}$ (B), both containing 0.1% formic acid as solvents.

The gradient was carried out as follows: 5%–100% B in 7 min, 1 min at 100% B and re-equilibration step at 5% B for 2 min.

The ELSD temperature was fixed at 45°C, with a gain of 9. PDA data were acquired from 190 to 500 nm, with a resolution of 1.2 nm. The sampling rate was set at 20 points/s. All data were processed with the MassLynx software (Waters, Milford, MA, United States).

#### 5.9.2 UHPLC-PDA-CAD-HRMS/MS analysis

Analyses were performed with a Vanquish Horizon (Thermo Scientific, Germany) equipped with a binary pump H, a dual split sampler HT and a Diode Array detector FG coupled to an Orbitrap Exploris 120 mass spectrometer (Thermo Scientific, Germany), and a Corona Veo RS Charged Aerosol Detector (CAD, Thermo Scientific, Germany). The Orbitrap employs a heated electrospray ionization source (H-ESI) with the following parameters: spray voltage: +3.5 kV; ion transfer tube temperature: 320.00°C; vaporizer temperature: 320.00°C; S-lens RF: 45 (arb units); sheath gas flow rate: 35.00 (arb units); Sweep Gas (arb): 1, and auxiliary gas flow rate: 10.00 (arb. units).

The mass analyzer was calibrated using a mixture of caffeine, methionine - arginine - phenylalanine - alanine - acetate (MRFA), sodium dodecyl sulfate, sodium taurocholate, and Ultramark 1621 in an acetonitrile/methanol/water solution containing 1% formic acid by direct injection. Control of the instruments was done using Thermo Scientific Xcalibur software v. 4.6.67.17. Full scans were acquired at a resolution of 30,000 fwhm (at *m/z* 200) and ${\text{MS}}^{2}$ scans at 15000 fwhm in the range of 100–1000 *m/z*, with 1 microscan, time (ms): 200 ms, an RF lens (%): 70; AGC target custom (Normalized AGC target (%): 300); maximum injection time (ms): 130; Microscans: 1; data type: profile; Usue EASY-IC(TM): ON. The Dynamic exclusion mode: Custom; Exclude after n times: 1; Exclusion duration (s): 5; Mass tolerance: ppm; low: 10, high: 10, Exclude isotopes: true. Appex detection: Desired Apex Window (%): 50. Isotope Exclusion: Assigned and unassigned with an exclusion window (*m/z*) for unassigned isotopes: 8. The Intensity threshold was set to 2.5E5. and a targeted mass exclusion list was used. The centroid data-dependent ${\text{MS}}^{2}$ (dd- ${\text{MS}}^{2}$ ) scan acquisition events were performed in discovery mode, triggered by Apex detection with a trigger detection (%) of 300 with a maximum injection time of 120 ms, performing 1 microscan. The top 3 abundant precursors (charge states 1 and 2) within an isolation window of 1.2 *m/z* were considered for MS/MS analysis. For precursor fragmentation in the HCD mode, a normalized collision energy of 15, 30, and 45% was used. Data was recorded in profile mode (Use EASY-IC(TM): ON)

#### 5.9.3 Generic reverse-phase UHPLC-HRMS/MS chromatographic method

The chromatographic separation was done on a Waters BEH C18 column ( 50×2.1 mm i.d., 1.7 μ m, Waters, Milford, MA) using a gradient as follows (time (min), %B):0.00, 5; 7, 100; 8.8, 100; 9.0, 5; 10, 5. The mobile phases were (A) water with 0.1% formic acid and (B) acetonitrile with 0.1% formic acid. The flow rate was set to 600 μ L/min, the injection volume was 2 μ L, and the column was kept at 40°C.

#### 5.9.4 Microfractionation on HPLC-DAD

*Chaetomium globosum* extracts (2.3 and 3.4 mg) were dissolved in 100 μ L of DMSO (LC-MS grade, Thermo Scientific, REF 85190) and injected in High-performance liquid chromatography HPLC-DAD-ESLD 1260 system (Agilent, Santa Clara, CA, United States) using Waters XBridge C18 column ( 250×4.6 mm i.d., 5 μ m, Waters, Milford, MA) for gradient optimization. The flow rate was set to 1000 μ L/min and the injection volume was 2 μ L. The mobile phases were (A) water with 0.1% formic acid and (B) acetonitrile with 0.1% formic acid. The optimized gradient was found to be: 15%–70% B in 44 min, 70%–100% B in 1 min and hold for 10 min at 100% B.

The optimized gradient was then geometrically transferred on a Waters XBridge C18 column ( 250×10 mm i.d., 5 μ m, Waters, Milford, MA) with a flowrate of 4.7 mL/min. The samples were injected (100 μ L) and the analysis was performed on HPLC 1200 system (Agilent, Santa Clara, CA, United States) equipped with a UV detector and a fraction collector. 1.5 mL fractions were collected directly in 96-well plate during the entire runs (165 fractions; equivalent to 2 × 96 deepwell plates).

### 5.10 Metabolomics analysis

#### 5.10.1 Data processing

The MS data were converted from.RAW (Thermo) standard data format to.mzXML format using the MSConvert software, part of the ProteoWizard package (Chambers et al., 2012). The converted files were treated using the MZmine software suite v. 4.0 (Schmid et al., 2023). The parameters were adjusted as follows: the centroid mass detector was used for mass detection with the noise level set to 1.0E4 for MS level set to 1, and to 0 for MS level set to 2. The ADAP chromatogram builder was used and set to a minimum group size of scans of 5, minimum group intensity threshold of 1.0E4, minimum highest intensity of 5.0E5 and *m/z* tolerance of 12 ppm. For chromatogram deconvolution, the algorithm used was the wavelets (ADAP) Myers et al. (2017). The intensity window S/N was used as S/N estimator with a signal to noise ratio set at 10, a minimum feature height at 5.0E5, a coefficient area threshold at 130, a peak duration ranges from 0.0 to 0.5 min and the RT wavelet range from 0.01 to 0.03 min. Isotopes were detected using the isotopes peaks grouper with a *m/z* tolerance of 12 ppm, a RT tolerance of 0.01 min (absolute), the maximum charge set at 2 and the representative isotope used was the most intense. Each feature list was filtered before alignment to keep only features with an associated ${\text{MS}}^{2}$ scan and a RT between 0.5 and 7.0 min using the feature filtering. At this step, feature tables and spectra were exported using the “export to GNPS”.

#### 5.10.2 GNPS ion identity networking

The resulting filtered list was subjected to Ion Identity Networking (Schmid et al., 2021) starting with the metaCorrelate module (RT tolerance, 0.10 min; minimum height, 1.0E5; Intensity correlation threshold 1.0E5 and the Correlation Grouping with the default parameters). This was followed by the Ion identity networking (*m/z* tolerance, 8.0 ppm; check: one feature; minimum height: 1.0E5, annotation library [maximum charge, 2; maximum molecules/cluster, 2; Adducts ( ${\left[\text{M}+\text{H}\right]}^{+}$ , ${\left[\text{M}+\text{Na}\right]}^{+}$ , ${\left[\text{M}+\text{K}\right]}^{+}$ , ${\left[\text{M}+{\text{NH}}_{4}\right]}^{+}$ , ${\left[\text{M}+2\text{H}\right]}^{2+}$ ), Modifications ( $\left[\text{M}-{\text{H}}_{2}\text{O}\right]$ , $\left[\text{M}-2{\text{H}}_{2}\text{O}\right]$ , $\left[\text{M}-{\text{CO}}_{2}\right]$ , [M+HFA] , [M+ACN] )], Annotation refinement (Delete small networks without major ion, yes; Delete networks without monomer, yes), Add ion identities networks (*m/z* tolerance, 8 ppm; minimum height, 1.0E5; Annotation refinement (Minimum size, 1; Delete small networks without major ion, yes; Delete small networks: Link threshold, 4; Delete networks without monomer, yes)) and Check all ion identities by MS/MS (*m/z* tolerance $\left({\text{MS}}^{2}\right)$ , 10 ppm; min-height (in ${\text{MS}}^{2}$ ), 1.0E3; Check for multimers, yes; Check neutral losses $\left({\text{MS}}^{1}\;\to \;{\text{MS}}^{2}\right)$ , yes) modules. The resulting aligned peak list was exported as a.mgf file for further analysis.

#### 5.10.3 MS/MS spectral organization

A molecular network was constructed from the.mgf file exported from MZmine, using the feature based molecular networking workflow (https://ccms-ucsd.github.io/GNPSDocumentation/) on the GNPS website (Aron et al., 2020). The precursor ion mass tolerance was set to 0.02 Da with an MS/MS fragment ion tolerance of 0.02 Da. A network was created where edges were filtered to have a cosine score above 0.7 and more than six matched peaks. The spectra in the network were then searched against GNPS’ spectral libraries. All matches between network and library spectra were required to have a score above 0.6, and at least three matched peaks.

The resulting Molecular networks (.graphml) were visualized and customized using Cytoscape suite v.3.10.2, an open-source software platform for complex network analysis and visualization.

#### 5.10.4 SIRIUS metabolite annotation

The SIRIUS.mgf file exported from MZmine (using the SIRIUS export module) that contains ${\text{MS}}^{1}$ and ${\text{MS}}^{2}$ information was processed with SIRIUS (v 5.8.6) (Dührkop et al., 2019). The molecular formula and metabolite database used for SIRIUS includes natural products (NPs) from LOTUS (Rutz et al., 2022) and the Dictionary of Natural Products (DNP). The parameters were set as follows: Possible ionizations: ${\left[\text{M}+\text{H}\right]}^{+}$ , ${\left[\text{M}+{\text{NH}}_{4}\right]}^{+}$ , ${\left[\text{M}-{\text{H}}_{2}\text{O}+\text{H}\right]}^{+}$ , ${\left[\text{M}+\text{K}\right]}^{+}$ , ${\left[\text{M}+\text{Na}\right]}^{+}$ , ${\left[\text{M}-4{\text{H}}_{2}\text{O}+\text{H}\right]}^{+}$ ; Instrument profile: Orbitrap; mass accuracy: 5 ppm for ${\text{MS}}^{1}$ and 7 ppm for ${\text{MS}}^{2}$ ; database for molecular formulas and structures: BIO and custom databases (LOTUS, DNP); maximum *m/z* to compute: 1000. ZODIAC was used to improve molecular formula prediction using a threshold filter of 0.99 (Ludwig et al., 2020). Metabolite structure prediction was performed with CSI: FingerID (Dührkop et al., 2015), and significance was computed with COSMIC (Hoffmann et al., 2021).

### 5.11 Antibacterial assay

Minimal inhibitory concentrations (MICs) on *S. aureus* strain Newman 1 were performed by two-fold serial dilutions in Mueller-Hinton Broth according to Clinical and Laboratory Standards Institute (CLSI) guidelines, at three different occasions. Extracts or fractions were suspended in DMSO (5 mg/mL) and their potential growth inhibition was assessed in Mueller-Hinton broth (one extract (4.5 μ L)/well (145.5 μ L medium). Wells containing DMSO (3% vol/vol) or vancomycin (10 mg/L final concentration) were used as vehicle and growth inhibition controls, respectively. To each well, $10^{4}$ – $10^{5}$ Colony Forming Units (CFU) of *S. aureus* strain Newman were added. After static incubation for 20 h at 37°C, absorbance at 600 nm was measured in each well using a Synergy H1 plate reader (Biotek^®^). Wells showing OD600 values equal to or below the vancomycin control were considered as growth inhibitory. Screenings were repeated at least once.

## Data Availability

The data presented in the study are deposited in the MassIVE repository, accession number MSV000097657.
