# Supplementary material for: Miniaturized high-throughput conversion of fungal strain collections into chemically characterized extract libraries for antimicrobial discovery
Source: Front Chem. 2025 Jul 2;13:1630332. doi: 10.3389/fchem.2025.1630332 (PMC12263559; doi:10.3389/fchem.2025.1630332)
Supplement: Supplementary file 1 [file DataSheet1.pdf]

Supplementary Material

1 SUPPLEMENTARY DATA

1.1 Original QuEChERS Protocols Adapted to 2 mL Scale

QuEChERS 1

- **Sample: Solvent**: 1mL:0.75mL CH_3CN
- **Matrices**: Vegetables
- **Salts**: 300 mg $MgSO_4$, 75 mg NaCl, 40 mg citrate acid, 4 mg NaH_2PO_4
- **d-SPE Sorbent (0.65 mL supernatant, Step 2)**: PSA 6.5 mg, C_{18} 16.3 mg, anhydrous $MgSO_4$ 98 mg
- **Reference**: (Wang et al., 2018)

QuEChERS 2

- **Sample: Solvent**: 1mL:0.75mL CH_3CN
- **Matrices**: Edible fungi
- **Salts**: 450 mg $MgSO_4$, 90 mg CH_3COONa
- **d-SPE Sorbent (0.65 mL supernatant, Step 2)**: PSA 6.5 mg, anhydrous $MgSO_4$ 32.5 mg
- **Reference**: (Zhu et al., 2021)

QuEChERS 3

- **Sample: Solvent**: 1mL:0.75mL EtOAc + 0.1% FA
- **Matrices**: Bacterial broth
- **Salts**: 300 mg $MgSO_4$, 75 mg NaCl
- **d-SPE Sorbent (0.65 mL supernatant, Step 2)**: C_{18} 9.8 mg, $MgSO_4$ 58.5 mg
- **Reference**: (Brosnan et al., 2014)

QuEChERS 4

- **Sample: Solvent**: 1mL:0.75mL CH_3CN + 0.1% FA
- **Matrices**: Food sample
- **Salts**: 300 mg Na_2SO_4, 75 mg NaCl, 75 mg $Na_3Cit \cdot 2\,H_2O$, 38 mg Na_3Cit dibasic sesquihydrate
- **d-SPE Sorbent (0.65 mL supernatant, Step 2)**: Na_2SO_4 98 mg, PSA 5.4 mg, C_{18} 16.3 mg, $MgSO_4$ 58.5 mg
- **Reference**: (Desmarchelier et al., 2018)

1.2 Original QuEChERS Mixture Adapted to 25 x 2 mL wells

QuEChERS 1:

Step 1: 419 mg of the following mixture was added per well.

7.5 *g* $MgSO_4$, 1.9 *g* NaCl, 1.0 g citric acid, 100 mg NaH_2PO_4.

Step 2: 123 mg of the following mixture was added per well.

PSA 162.5 mg, C18 407.5 mg, GCB 62.5 mg, 2.5 g anhydrous $MgSO_4$

QuEChERS 2:

Step 1: 540 mg of the following mixture was added per well.

11.3 *g* $MgSO_4$, 2.3 *g* CH_3COONa

Step 2: 39 mg of the following mixture was added per well.

PSA 162.5 mg, 812.5 mg anhydrous $MgSO_4$

QuEChERS 3:

Step 1: 375 mg of the following mixture was added per well.

7.5 *g* $MgSO_4$, 1.9 *g* NaCl

Step 2: 68 mg of the following mixture was added per well.

C18 245 mg, 1.5 *g* $MgSO_4$

QuEChERS 4:

Step 1: 488 mg of the following mixture was added per well.

7.5 *g* Na_2SO_4, 1.9 *g* NaCl, 1.9 *g* $Na_3Cit \cdot 2\,H_2O$, 950 mg Na_3Cit dibasic sesquihydrate

Step 2: 120 mg of the following mixture was added per well.

2.5 *g* Na_2SO_4, PSA 135 mg, C18 407.5 mg

2 SUPPLEMENTARY TABLES AND FIGURES

2.1 Tables

2.2 Figures

Table S1. Selected fungal strains (ABO-P09) – Part 1

Index	Mycotheca ID	Species Name	Date of Sampling	Host
1	6	*Fusarium culmorum*	1989	Potato
2	10	*Epicoccum nigrum*	2004	Grapevine
3	17	*Fusarium poae*	1993	Barley
4	24	*Oculimacula yallundae*	-	Wheat
5	34	*Boeremia exigua*	1989	Potato
6	38	*Laxitextum incrustatum*	1990	Potato
7	45	*Fusarium oxysporum*	2000	Tomato seed
8	68	*Dactylonectria alcacerensis*	2004	Grapevine
9	83	*Didymella arachidicola*	1992	Pea
10	93	*Eutypa lata*	2004	Grapevine
11	99	*Clonostachys rosea*	2004	Grapevine
12	123	*Verticillium dahliae*	2011	Artemisia stem
13	159	*Verticillium lateritium*	1993	Wheat
14	264	*Diplocarpon rosae*	2013	Rose leaf
15	362	*Cylindrocarpon destructans*	2000	Strawberry
16	440	*Ceratocystis fimbriata*	2013	Grapevine grafting point
17	445	*Paraconiothyrium sporulosum*	1980	Raspberry stem
18	447	*Phomopsis mali*	1967	Apple tree
19	448	*Pseudopyrenochaeta lycopersici*	-	Tomato root
20	461	*Coryneum microsticum*	1973	Rose stem
21	482	*Fomitiporia mediterranea*	-	Grapevine
22	483	*Stemphylium botryosum*	1985	Asparagus stem
23	488	*Verticillium alboatrum*	1981	Strawberry root
24	498	*Trametes versicolor*	1963	-
25	499	*Stereum purpurem*	1962	-
26	511	*Fusarium poae*	1977	Wheat
27	515	*Trichoderma viride*	1975	Elm
28	516	*Truncatella angustata*	1975	Grapevine
29	532	*Diaporthe oncostoma*	1981	Japanese maple
30	542	*Gliocladium virens*	1979	Sclerotinia minor
31	562	*Neofusicoccum parvum*	1988	Sequoia branches
32	566	*Stereum purpureum*	1980	Pear tree
33	569	*Nigrospora oryzae*	1982	Maize
34	579	*Dactylonectria alcacerensis*	2013	Grapevine grafting point
35	592	*Penicillium rubens*	1989	Sclerotium
36	594	*Sarocladium strictum*	2001	-
37	597	*Trichoderma pseudokoningii*	-	CBS collection
38	602	*Verticillium lecanii*	1989	Thrips
39	623	*Monilinia laxa*	1992	Apricot tree
40	629	*Alternaria alternata*	2002	Sunflower leaf
41	636	*Fusarium decemcellulare*	2006	-
42	685	*Chalaropsis thielavioides*	1995	Carrot root
43	734	*Phoma macdonaldii*	1997	Sunflower stem
44	818	*Armillaria bulbosa*	1987	CBS collection
45	823	*Armillaria mellea*	1994	Maple

Table S2. Selected fungal strains (ABO-P09) – Part 2

Index	Mycotheca ID	Species Name	Date of Sampling	Host
46	831	*Aspergillus pseudoglaucus*	1975	Cellar dust
47	835	*Beauveria pseudobassiana*	1971	Insect
48	856	*Ciboria viridifusca*	1996	CBS collection
49	872	*Cunninghamella elegans*	1992	CBS collection
50	880	*Eremascus fertilis*	1978	CBS collection
51	893	*Gnomonia sanguisorbae*	1981	CBS collection
52	900	*Hohenbuehelia angustata*	1998	CBS collection
53	907	*Phaeoacremonium inflatipes*	-	CBS collection
54	911	*Kloeckera apiculata*	1972	CBS collection
55	912	*Laetiporus sulphureus*	1993	CBS collection
56	930	*Nematospora coryli*	-	CBS collection
57	934	*Phialophora gregata*	1999	Soja
58	938	*Pholiota lucifera*	1994	-
59	945	*Monilinia fructigena*	2014	Grapevine berry
60	948	*Eurotium chevalieri*	1969	-
61	953	*Pycnoporus sanguineus*	1987	-
62	963	*Rhodotorula mucilaginosa*	1969	-
63	971	*Armillaria mellea*	1979	CBS collection
64	980	*Serpula lacrymans*	2000	Cellar wall
65	983	*Sporidiobolus salmonicolor*	1969	-
66	989	*Thamnidium ctenidium*	1972	-
67	995	*Waitea circinata*	1990	-
68	1026	*Botryosphaeria obtusa*	2008	Grapevine
69	1032	*Chaetomium globosum*	2008	Grapevine
70	1033	*Cadophora luteo-olivacea*	2008	Grapevine
71	1036	*Truncatella angustata*	2008	Grapevine
72	1044	*Phialophora fastigiata*	2008	Grapevine
73	1273	*Hypholoma fasciculare*	2015	Rain
74	1275	*Thysanophora penicillioides*	2015	Rain
75	1276	*Leptosphaerulina chartarum*	2015	Rain
76	1338	*Cladosporium cladosporioides*	2016	Fungal community
77	1677	*Curvularia trifolii*	2019	Turf
78	1688	*Mortierella elongata*	2019	Turf
79	1691	*Laetisaria fuciformis*	2019	Turf
80	1693	*Eurotium amstelodami*	2019	Juniper berry
81	1833	*Lasiodiplodia theobromae*	2019	Avocado
82	1854	*Penicillium expansum*	2019	Raspberry
83	1860	*Trichoderma citrinoviride*	2019	Mango
84	1871	*Penicillium solitum*	2019	Orange
85	1874	*Scopulariopsis brevicaulis*	2019	Cheese
86	2623	*Pleurotus ostreatus*	2019	Oyster mushroom
87	2636	*Exophiala oligosperma*	2020	-
88	2992	*Botrytis cinerea*	-	-
89	3320	*Syncephalastrum racemosum*	2021	Juniper berry
90	3390	*Simplicillium lamellicola*	2019	Rain

Table S3. Selected fungal strains (ABO-P26)

Index	Mycotheca ID	Species Name	Date of Sampling	Host
1	358	*Fusarium culmorum*	1981	Asparagus
2	1107	*Fusarium poae*	2006	Maize ear
3	530	*Fusarium thapsinum*	2006	-
4	561	*Fusarium nygamai*	2006	-
5	605	*Fusarium circinatum*	2006	-
6	617	*Fusarium lateritium*	2006	-
7	626	*Fusarium graminearum*	1993	Soja
8	1256	*Botrytis cinerea*	-	-
9	1880	*Fusarium equiseti*	2019	Banana stem
10	284	*Chaetomium globosum*	-	-
11	2941	*Chaetomium globosum*	2020	Wood
12	3514	*Botrytis cinerea*	2020	Rain
13	3512	*Coprinellus disseminatus*	2020	Rain
14	3513	*Trichoderma atroviride*	2020	Rain

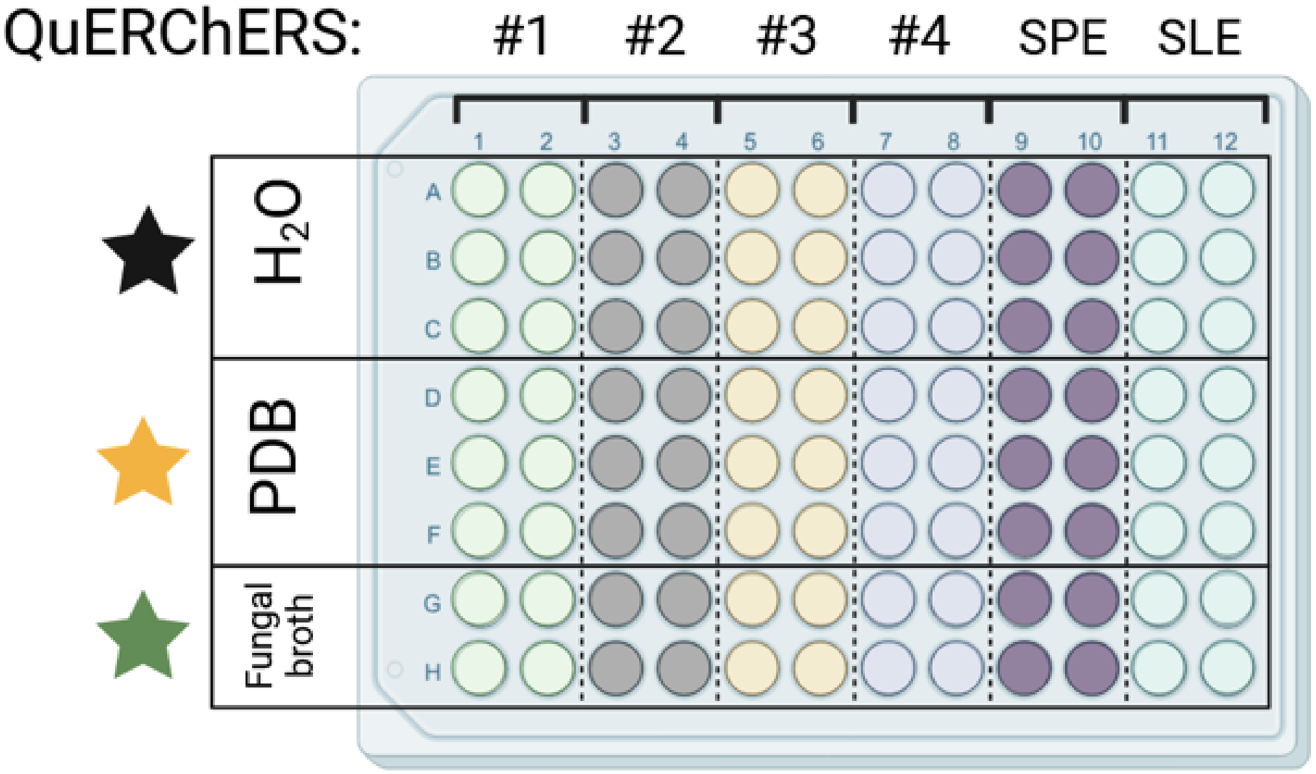

Figure S1: Plate scheme with the different extraction protocols (QuEChERS, SPE and SLE) as well as the different liquid matrices used (H_2O, PDB and fungal broth filtrate). Illustration was done with Biorender

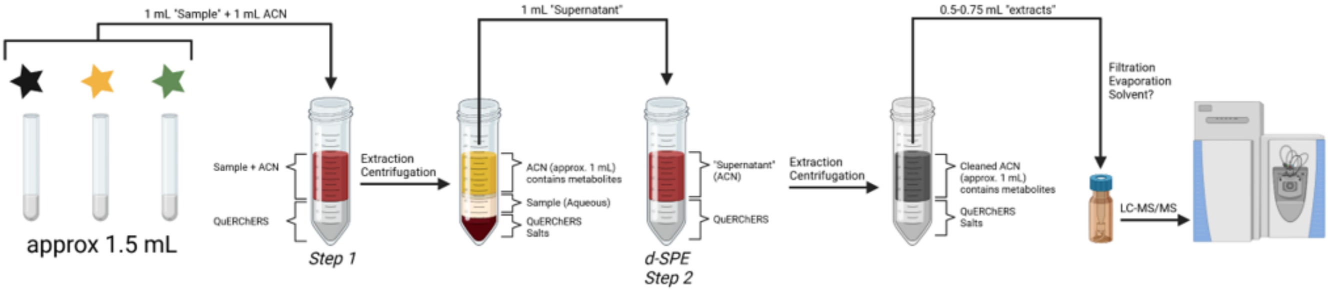

Figure S2: QuERChERS protocol adapted to 1.5 mL of liquid matrices (H_2O, PDB and fungal broth filtrate). Illustration was done with Biorender

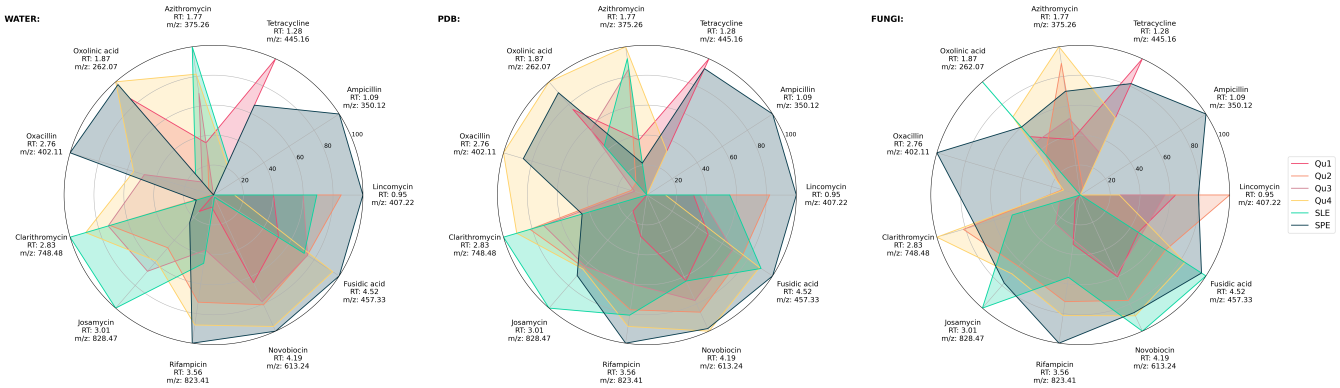

Figure S3: The results for the three liquid matrices (H_2O, PDB and fungal broth filtrate) are displayed in separated spider plot where antibiotic recovery rates, obtained for each extraction method, are displayed. Each antibiotic is displayed around the spider plot with its corresponding retention time (RT) and the m/z value used for quantification. For each combination (antibiotic:liquid matrix), data were normalized per antibiotic and the mean value is displayed. Each color corresponds to an extraction method: QuEChERS (Qu1-4), Supported Liquid Extraction (SLE), and Solid Phase Extraction (SPE).

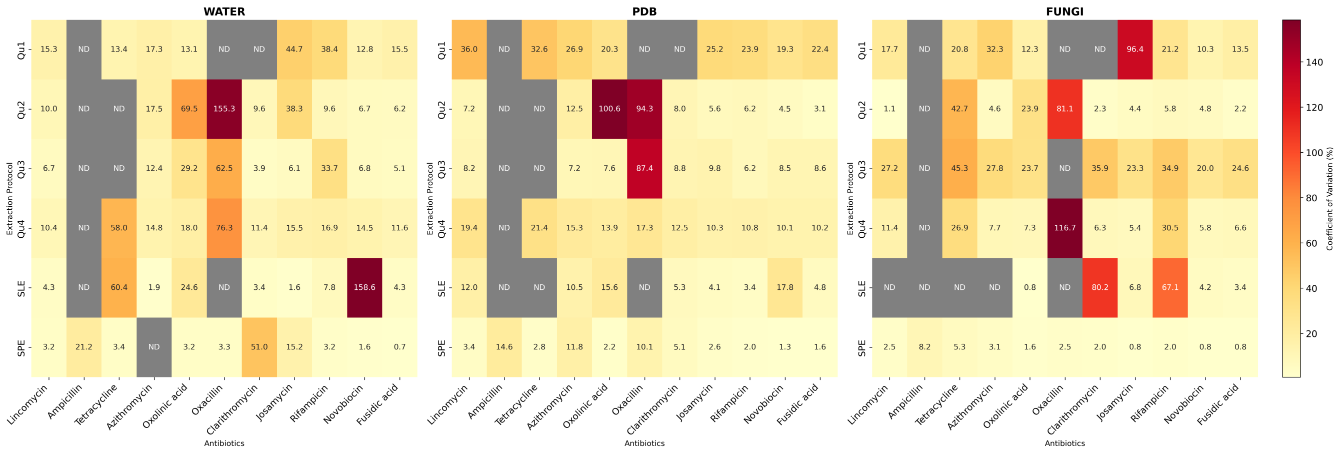

Figure S4: Variability expressed as the coefficient of variation for each extraction protocol and for the three liquid matrices (H_2O, PDB and fungal broth filtrate) are displayed as heat-maps. The values express the coefficient of variation in %. ND: Not detected.

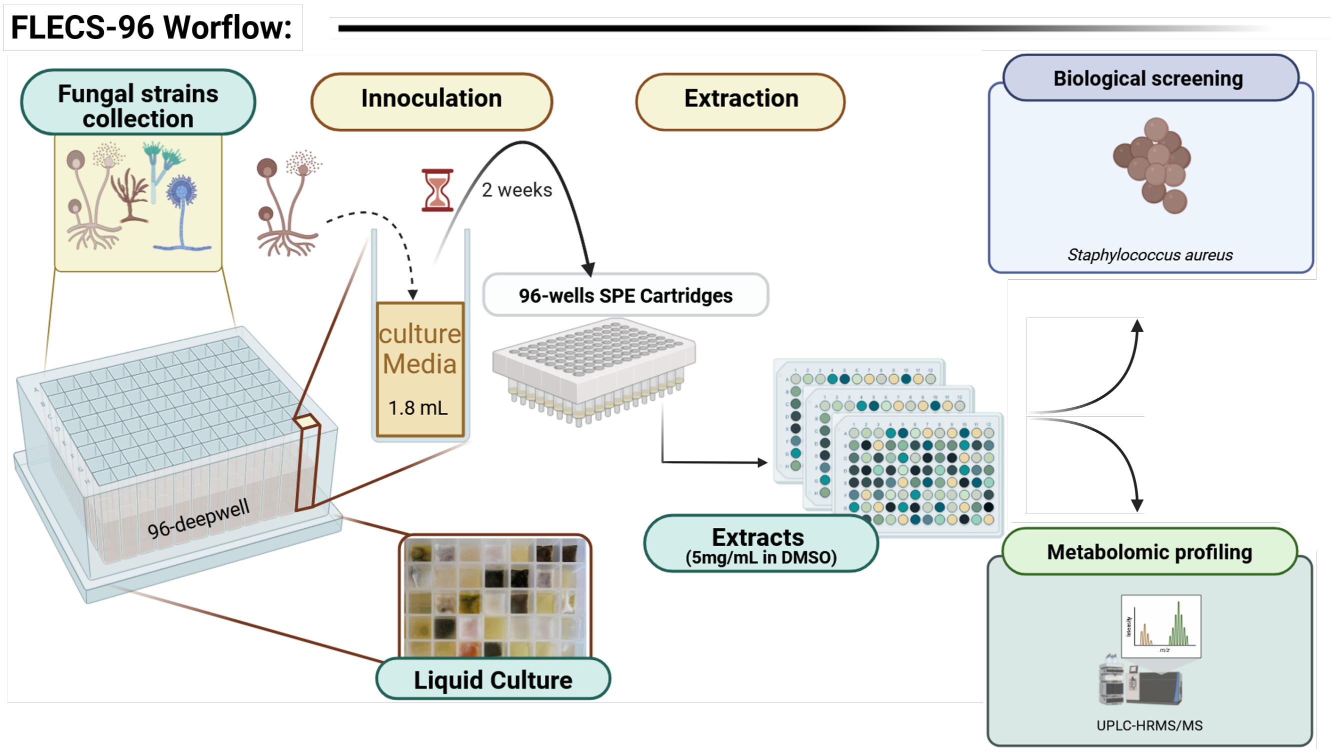

Figure S5: The FLECS-96 workflow is presented here as a scheme, from strains collection to the obtained extracts. The later, re-suspended in DMSO at 5mg/mL are suitable for antimicrobial assays against *S.aureus* and metabolomic profiling.

BEFORE:

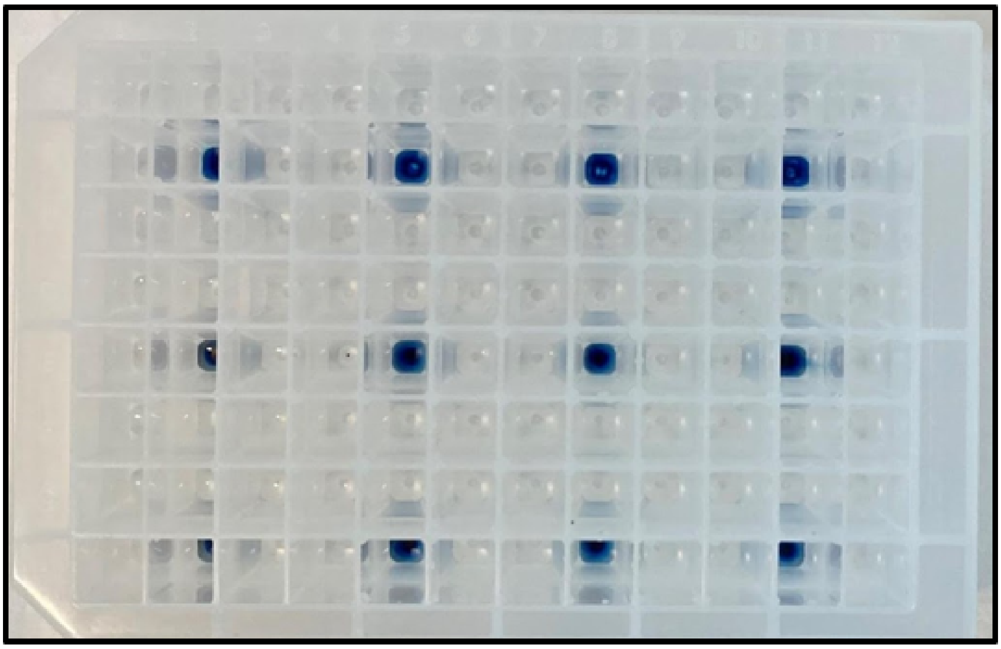

AFTER:

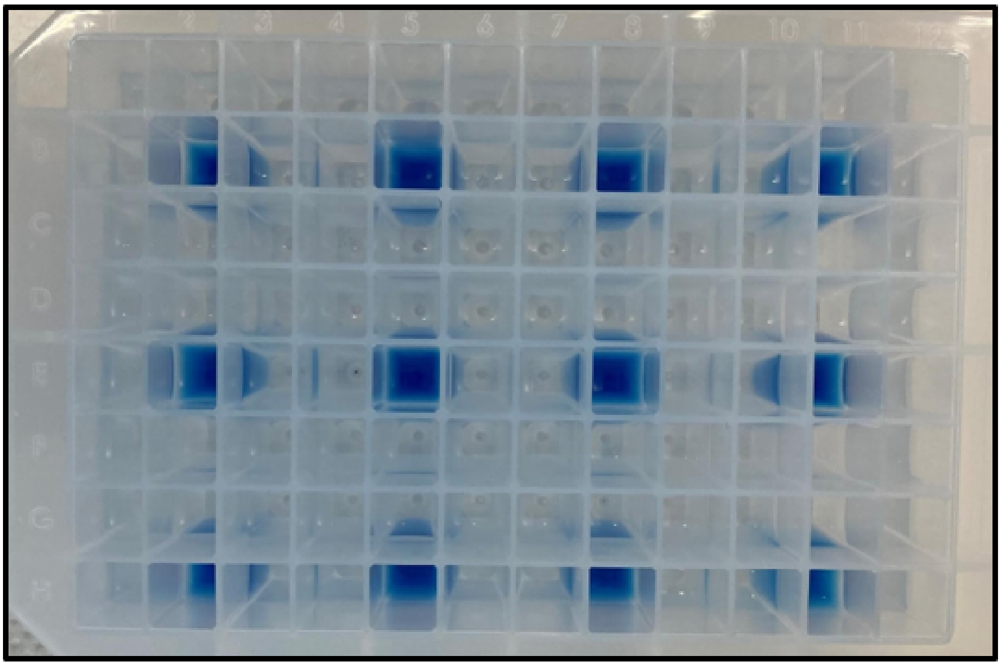

Figure S6: Assessment of Well-to-Well Contamination Using Blue Dye Diffusion: Representative before-and-after images showing the addition of blue dye to selected wells of a 96-deepwell microplate. The spread of dye into adjacent wells was monitored by visual inspection to evaluate potential cross-contamination between neighboring wells.

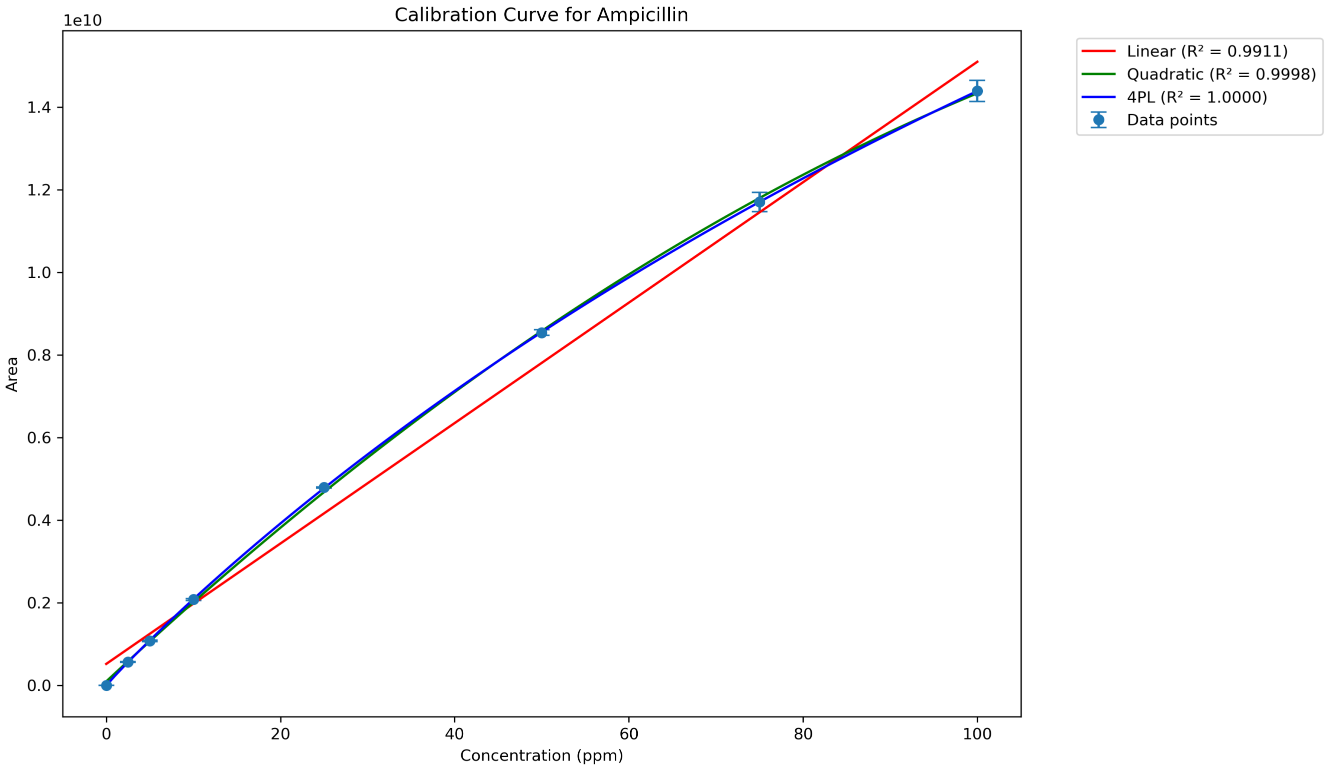

Figure S7: Calibration Curve Ampicillin

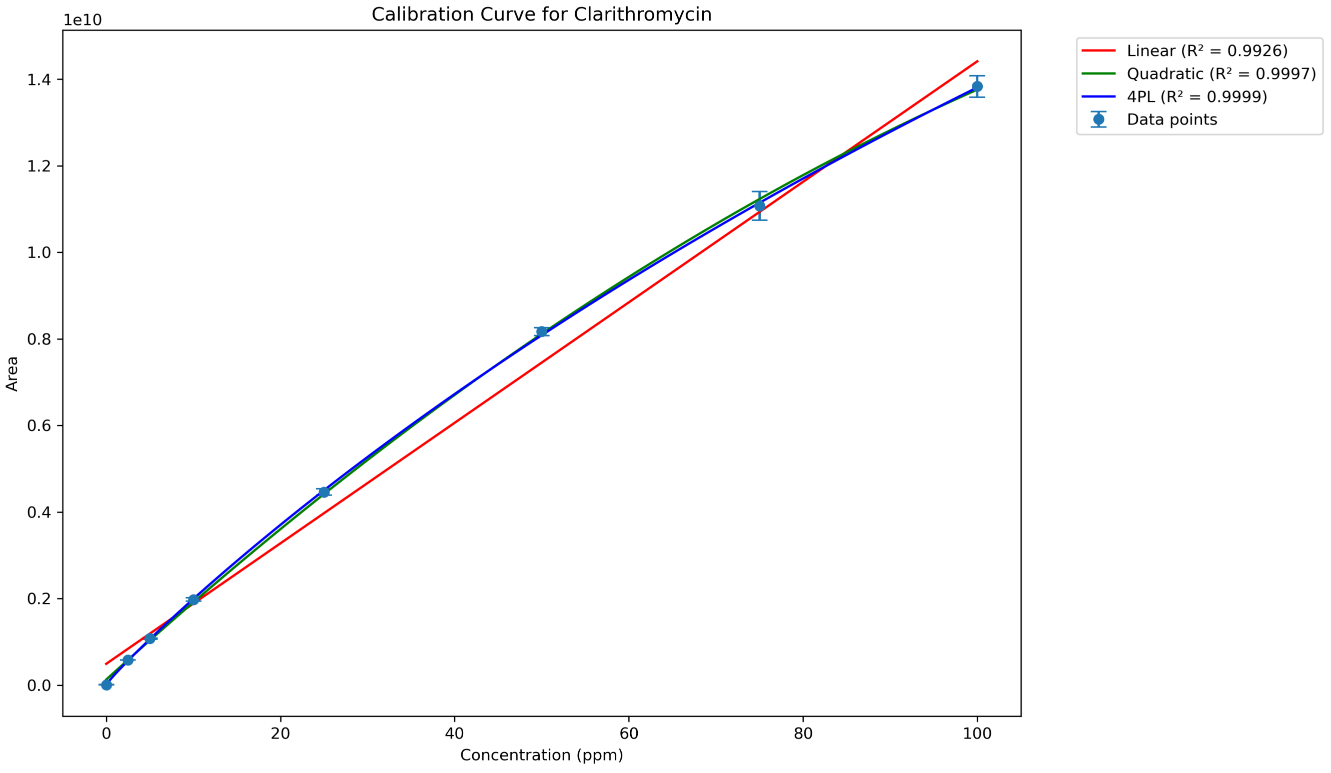

Figure S8: Calibration Curve Clarithromycin

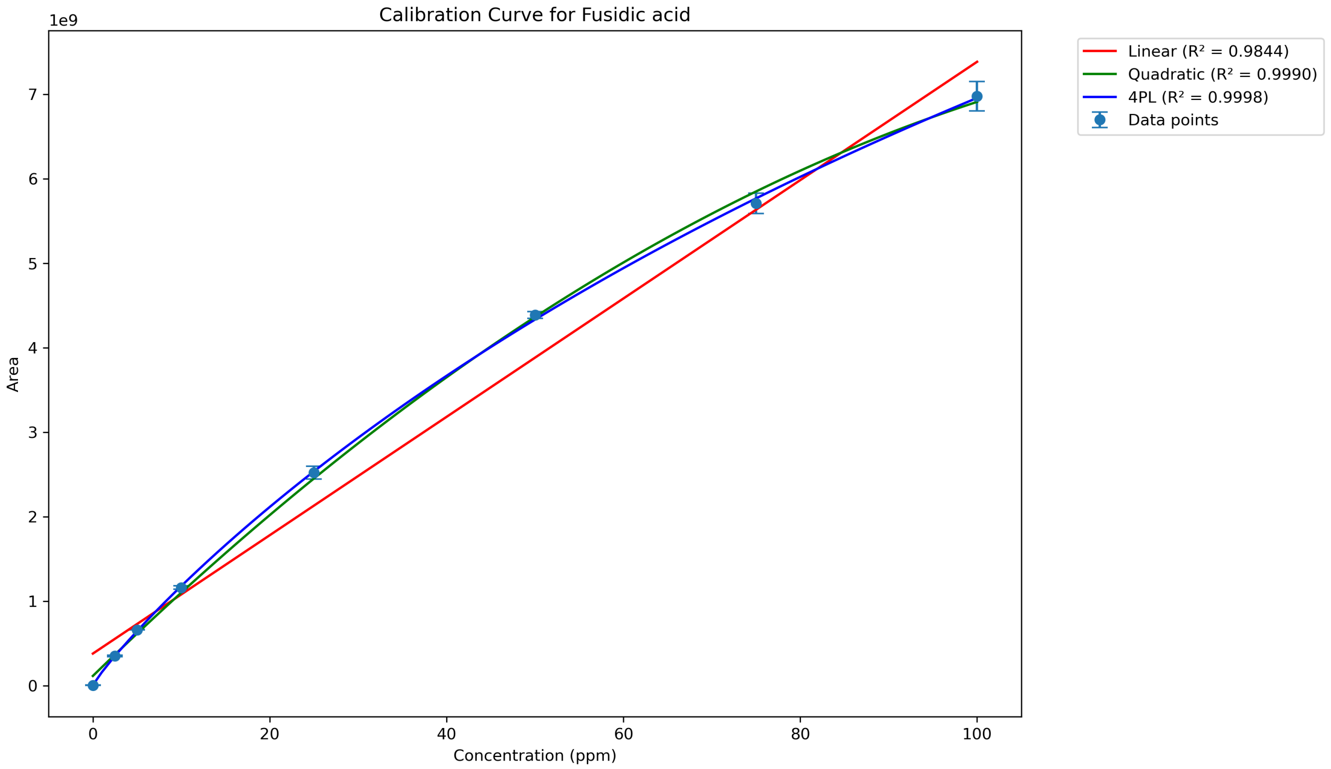

Figure S9: Calibration Curve Fusidic Acid

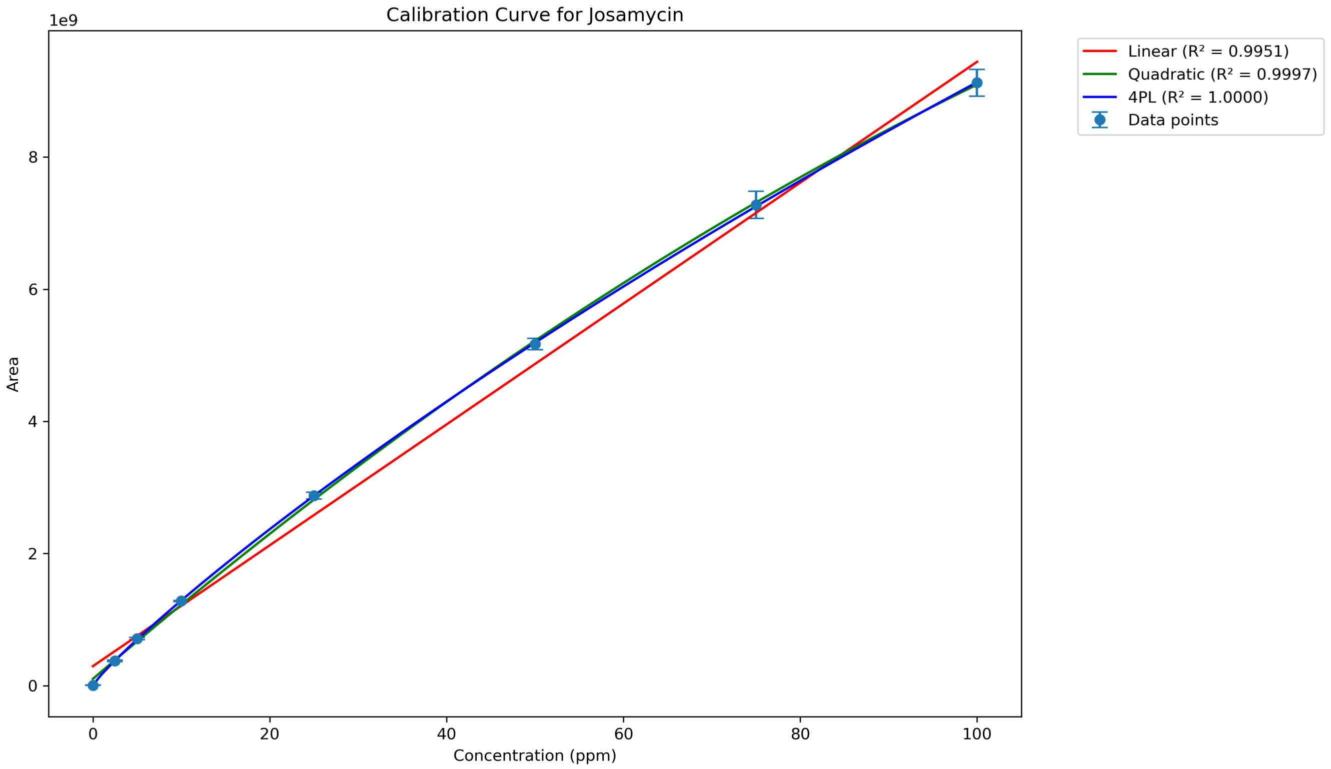

Figure S10: Calibration Curve Josamycin

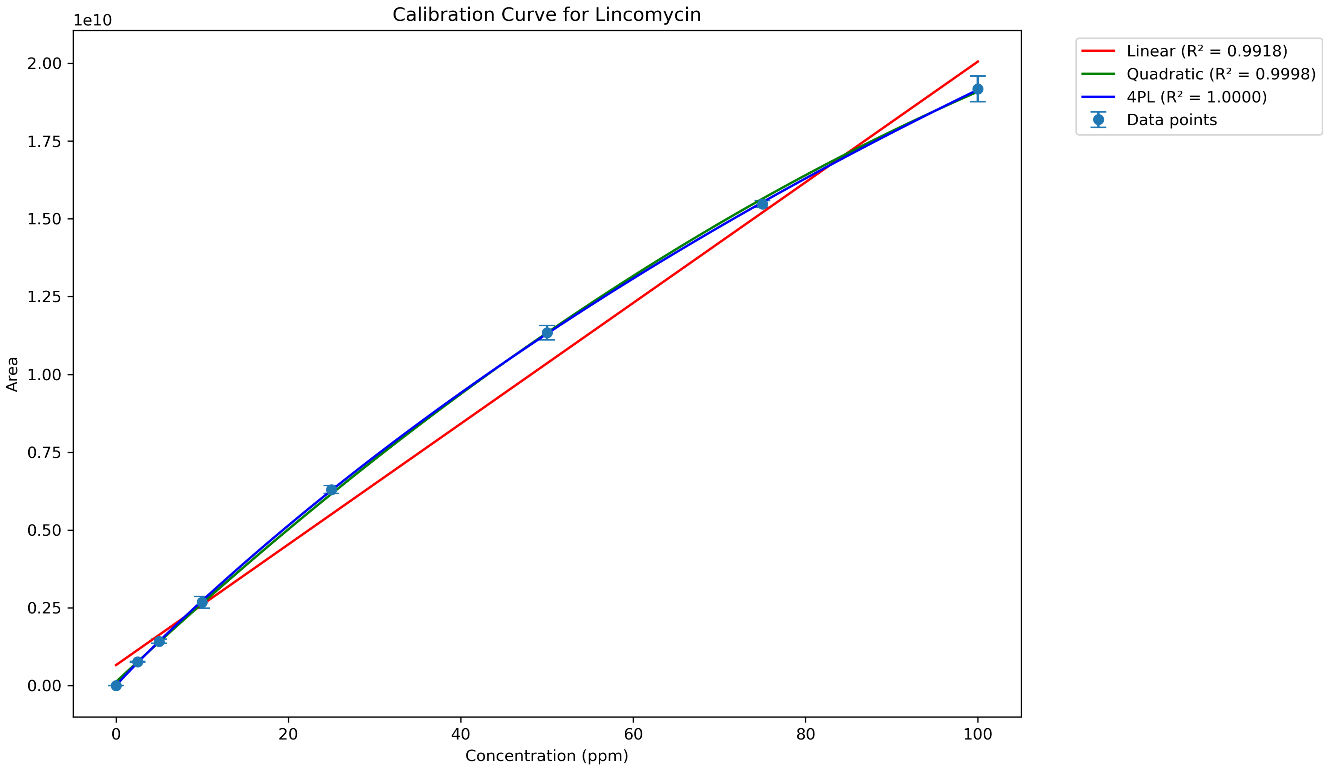

Figure S11: Calibration Curve Lincomycin

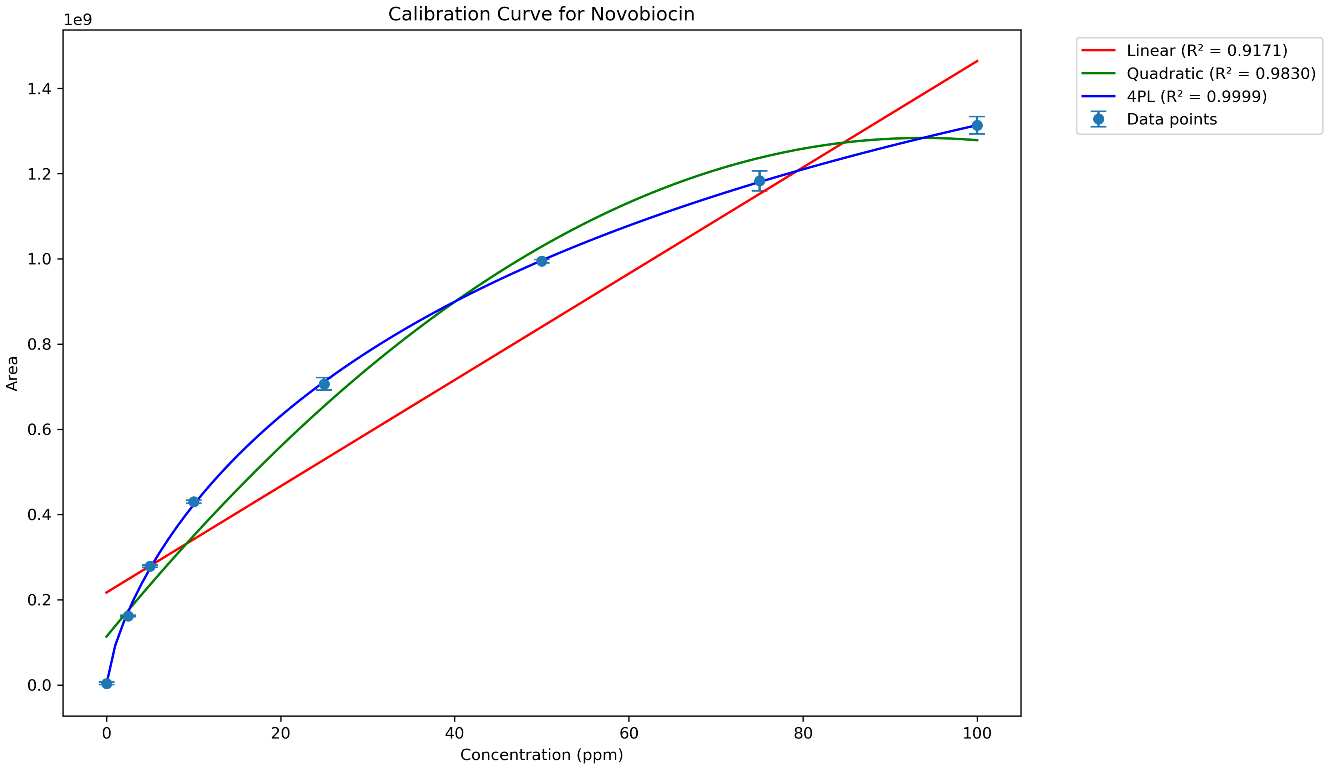

Figure S12: Calibration Curve Novobiocin

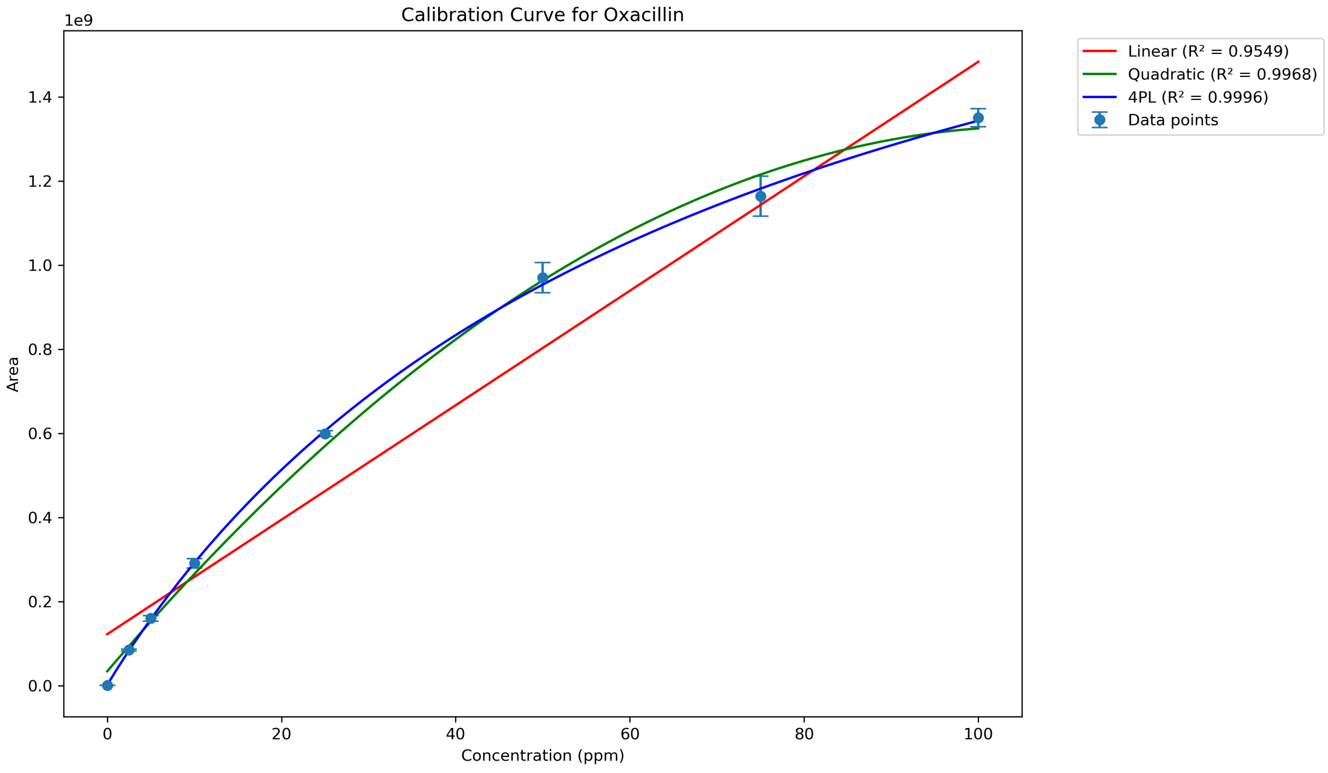

Figure S13: Calibration Curve Oxacillin

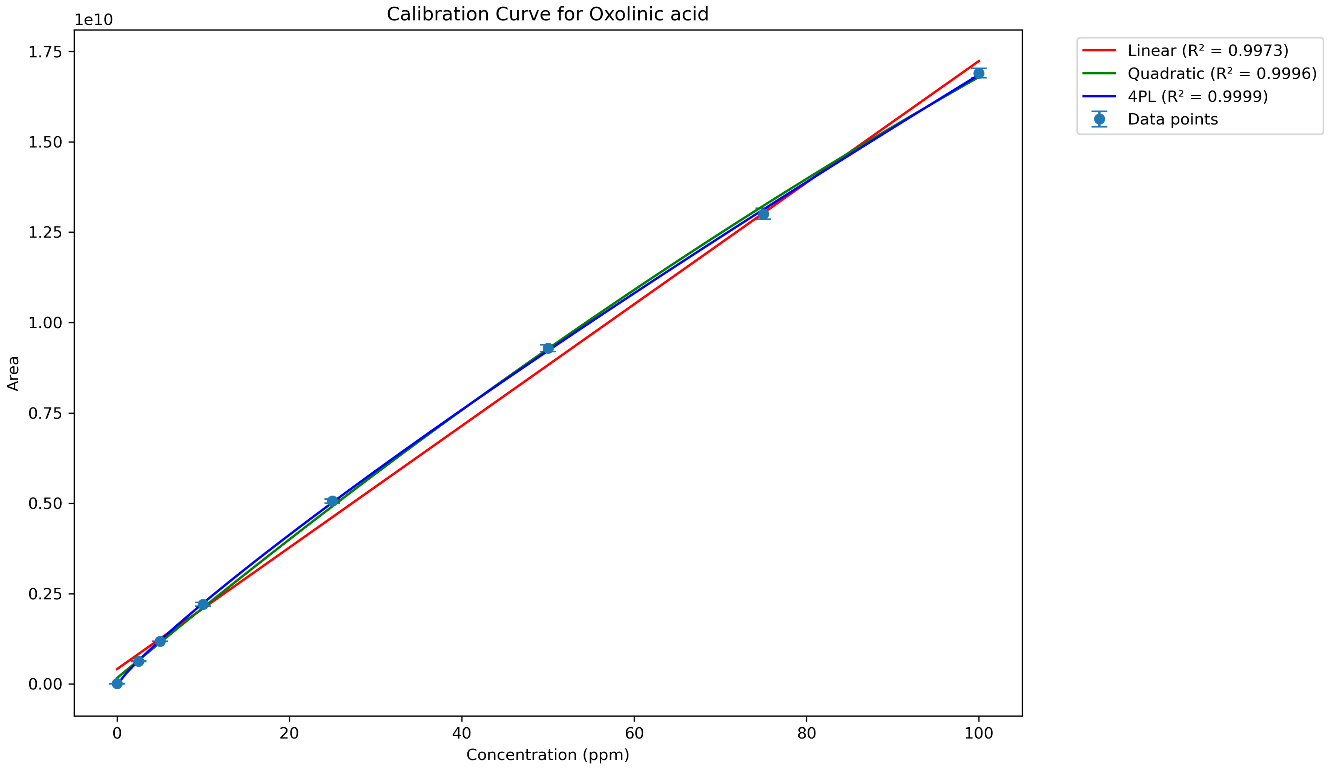

Figure S14: Calibration Curve Oxolinic Acid

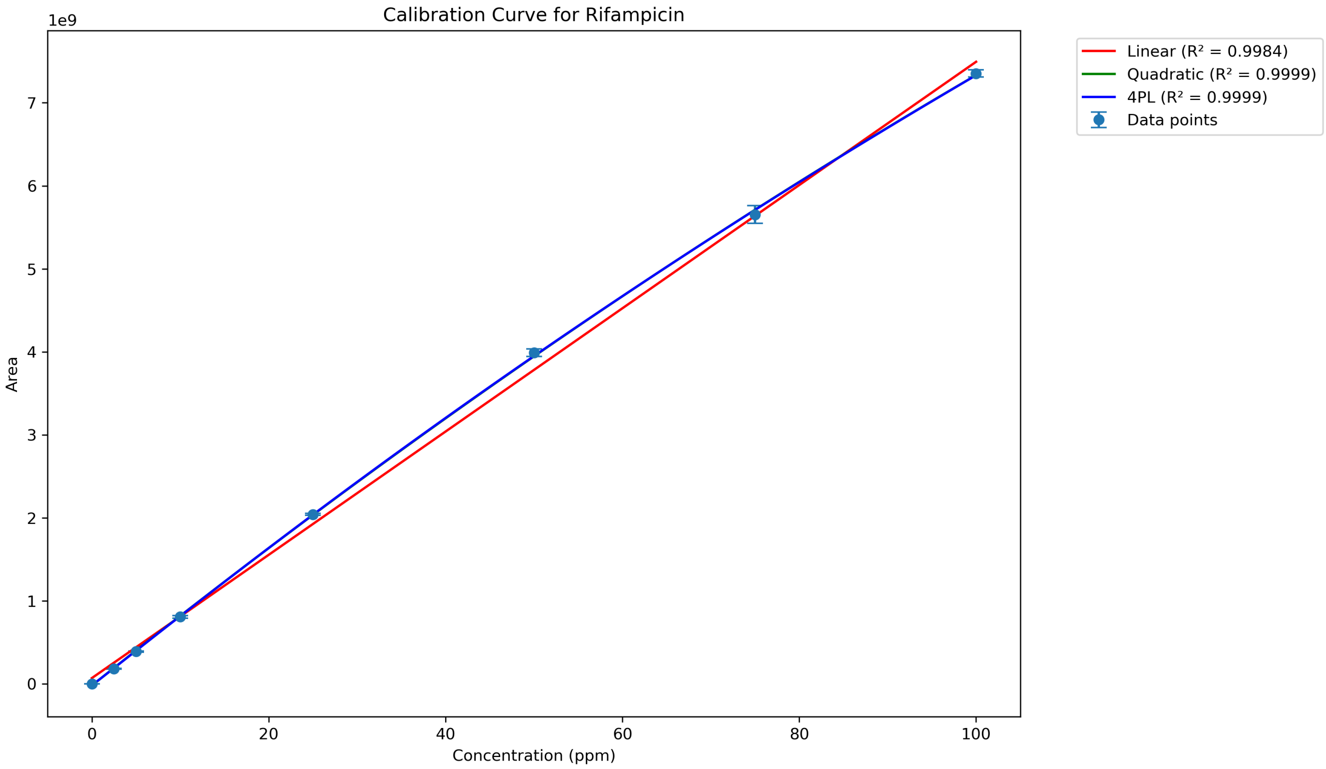

Figure S15: Calibration Curve Rifampicin

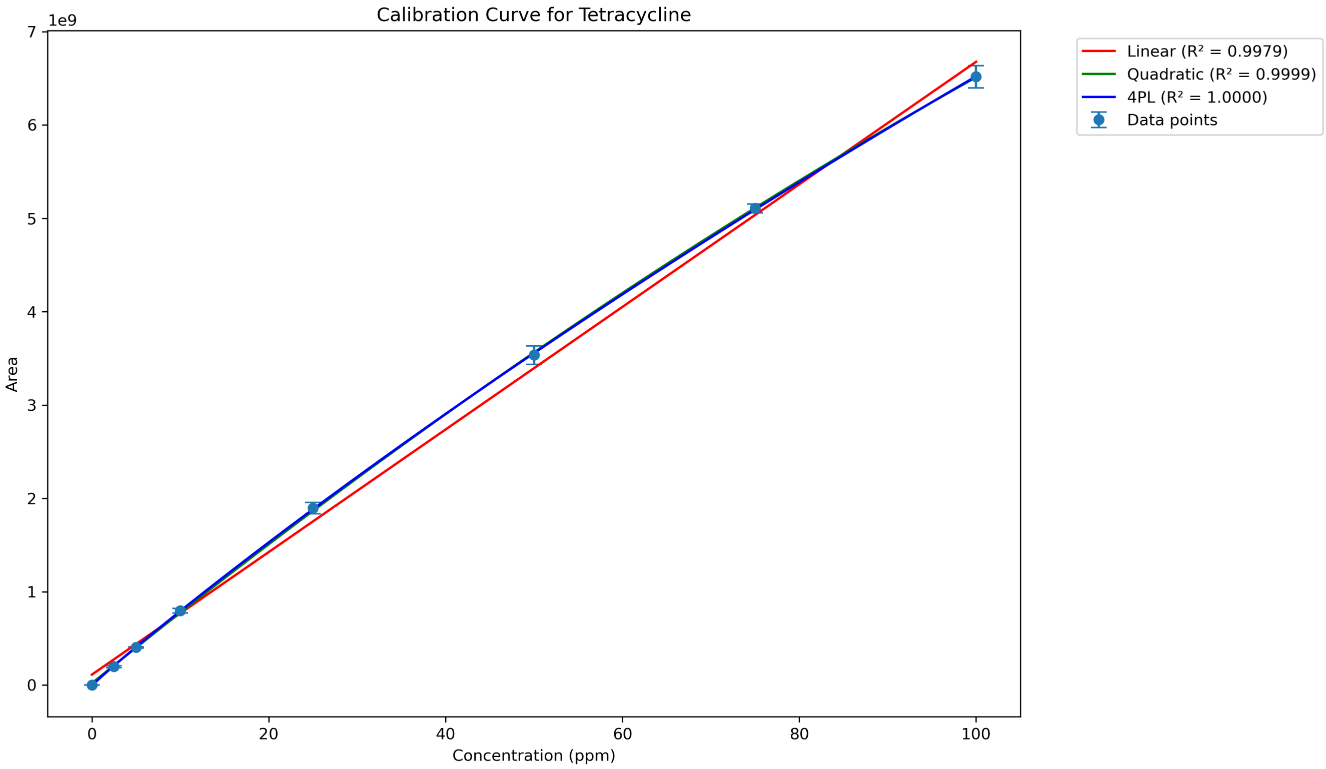

Figure S16: Calibration Curve Tetracycline

REFERENCES

Brosnan, B., Coffey, A., Arendt, E. K., and Furey, A. (2014). The QuEChERS approach in a novel application for the identification of antifungal compounds produced by lactic acid bacteria cultures. *Talanta* 129, 364–373. doi:10.1016/j.talanta.2014.05.006

Desmarchelier, A., Fan, K., Minh Tien, M., Savoy, M.-C., Tarres, A., Fuger, D., et al. (2018). Determination of 105 antibiotic, anti-inflammatory, antiparasitic agents and tranquilizers by LC-MS/MS based on an acidic QuEChERS-like extraction. *Food Additives & Contaminants: Part A* 35, 647–661. doi:10.1080/19440049.2018.1429677

Wang, W., Liao, Y., Chen, R., Hou, Y., Ke, W., Zhang, B., et al. (2018). Chlorinated Azaphilone Pigments with Antimicrobial and Cytotoxic Activities Isolated from the Deep Sea Derived Fungus Chaetomium sp. NA-S01-R1. *Marine Drugs* 16, 61. doi:10.3390/md16020061

Zhu, S., Gao, M., Tian, S., Bu, Y., Cui, H., Gan, Z., et al. (2021). Simultaneous Determination of 19 Antibiotics and 19 Anthelmintics Residues in Edible Fungi by UHPLC-MS/MS in Combination with QuEChERS Method. *Food Analytical Methods* 14, 1278–1288. doi:10.1007/s12161-020-01949-3
